# Progress and challenges in the synthesis of sequence controlled polysaccharides

**DOI:** 10.3762/bjoc.17.129

**Published:** 2021-08-05

**Authors:** Giulio Fittolani, Theodore Tyrikos-Ergas, Denisa Vargová, Manishkumar A Chaube, Martina Delbianco

**Affiliations:** 1 Department of Biomolecular Systems, Max Planck Institute of Colloids and Interfaces, Am Mühlenberg 1, 14476 Potsdam, Germany; 2 Department of Chemistry and Biochemistry, Freie Universität Berlin, Arnimallee 22, 14195 Berlin, Germany

**Keywords:** enzymes, glycans, polysaccharides, synthesis, well-defined polymers

## Abstract

The sequence, length and substitution of a polysaccharide influence its physical and biological properties. Thus, sequence controlled polysaccharides are important targets to establish structure–properties correlations. Polymerization techniques and enzymatic methods have been optimized to obtain samples with well-defined substitution patterns and narrow molecular weight distribution. Chemical synthesis has granted access to polysaccharides with full control over the length. Here, we review the progress towards the synthesis of well-defined polysaccharides. For each class of polysaccharides, we discuss the available synthetic approaches and their current limitations.

## Introduction

Polysaccharides are an abundant class of natural polymers that play important roles in the biosphere by structurally supporting plants, providing energy to animals, and regulating a variety of biological processes [1]. Their versatility and diversity result in a wide range of properties exploited for commercial purposes. Chemical modifications permit to broaden their applications even further. Additionally, polysaccharides can be converted into useful chemicals upon biodegradation [2–3], contributing to the sustainable development of future materials.

Despite polysaccharides’ utility, there are limitations associated with their exploitation. Extraction from natural sources is laborious, low yielding, and provides heterogeneous mixtures that hamper characterization, reproducibility, and quality control. Small contaminations can heavily affect the polysaccharides’ material and biological properties. This issue became dramatic when contaminated batches of heparin caused many deaths during the so called “heparin crisis” in 2008 [4].

In addition, the heterogeneity of naturally sourced samples poses a severe bottleneck to the molecular characterization of polysaccharides that dwarfs in comparison to other biomolecules like peptides and nucleic acids. Establishing structure–property correlations is key to understanding polysaccharides’ function in nature and translate this knowledge into tailor-made materials.

**Figure 1:**
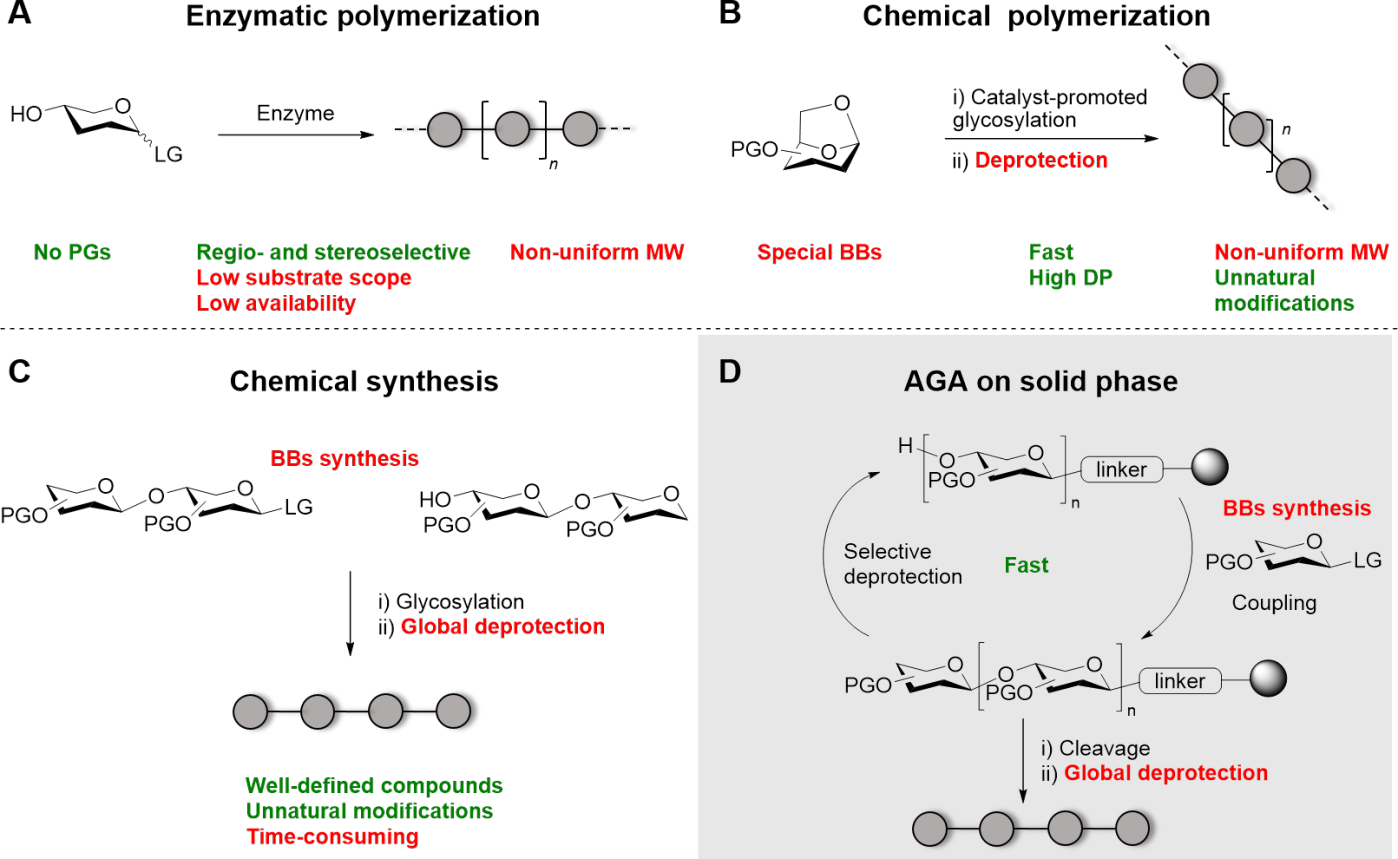
Overview of the methods available for the synthesis of polysaccharides. For each method, advantages (green) and disadvantages (red) are highlighted. Polymerization techniques (A and B) generate mixtures of compounds with non-uniform distribution of molecular weight (MW). In contrast, chemical synthesis (C) produces well-defined polysaccharides, but it is time-consuming. Automated Glycan Assembly (AGA) increases the efficiency of chemical synthesis by automating cycles of coupling and deprotection on a solid support (D).

Synthesis (i.e., chemical, enzymatic, or through polymerization) offers the opportunity to access polysaccharides with well-defined composition, length, and substitution. These compounds are ideal probes to study polysaccharides at the molecular level and identify structure–property correlations. Access to synthetic polysaccharides facilitated the correlation of chemical structure with molecular conformations [5–6], intermolecular interactions [7–8], and biological response [9]. Nevertheless, the complexity and diversity of polysaccharides makes synthetic processes extremely laborious and time consuming. Several aspects are crucial to plan a successful polysaccharide synthesis. Properly designed starting materials and/or catalysts are required to ensure regio- and stereocontrol during glycosidic bond formation. Control over the polysaccharide length (degree of polymerization, DP) and regioselective insertion of modifications or branches are additional challenges. Three main approaches are available (Figure 1): A) enzymatic polymerization [10–17], B) chemical polymerization [18], C) chemical synthesis.

The use of enzymes has undeniable advantages because it offers the possibility to use unprotected sugars as substrates and guarantees remarkable control of the regio- and stereoselectivity during glycosylation. Mono- or oligosaccharides bearing a reactive leaving group (LG, e.g*.,* phosphate, fluoride, nucleotide) are polymerized by the enzyme to form the desired polysaccharide (Figure 1A). Several classes of enzymes are available, including hydrolases, phosphorylases, sucrases, glycosyltransferases, and glycosynthases [19–22]. An excellent overview of the enzymes available for polysaccharide synthesis and their mode of action was recently published [11]. Despite the numerous advantages of this approach, limited enzyme availability as well as their high specificity narrowed the substrate scope. Generally, the highly specific enzyme reactive site tolerates only small modifications, hampering the formation of unnatural polymers. Low glycosylation yields and product hydrolysis represent additional hurdles associated with enzymatic synthesis of polysaccharides [23]. With this approach, homopolymers are often obtained as non-uniform samples, because the enzymes cannot distinguish between acceptors with different lengths in the reaction mixture.

Chemical polymerization is often performed by ring-opening of the respective anhydrosugar (Figure 1B) or polycondensation reactions. This approach is highly valuable to generate long chains in short time, allowing for the introduction of unnatural modifications. However, the control over the length and substitution pattern remains poor. To ensure good regio- and stereoselectivity, the starting material, often a polycyclic compound, has to be designed with suitable protecting groups (PGs). These structures can be quite challenging to prepare. Moreover, problems can occur during the removal of the PGs, since partially deprotected compounds can generate insoluble aggregates [24]. Incomplete deprotection and residual PGs can largely affect the properties of the obtained polymer.

Chemical synthesis provides compounds with well-defined length and substitution pattern (Figure 1C), but requires a substantial synthetic effort. For this reason, only few examples of long polysaccharides prepared by chemical synthesis are available. Properly designed building blocks (BBs) are needed, often prepared following numerous synthetic steps. In general, BBs are equipped with a reactive anomeric LG to allow for glycosylation and suitable PGs to ensure regio- and stereocontrol [25]. Even though, in most cases, BB preparation follows straightforward protection/deprotection strategies, the low selectivity and yield of certain transformations [26–27] can limit the scope of this approach. The desired polysaccharide is generally assembled following a linear or a convergent approach. In the former, the desired BBs are added sequentially to the growing polysaccharide chain via a series of glycosylation reactions. In contrast, a convergent approach (also known as fragment coupling) allows connecting pre-assembled oligosaccharide blocks. To decrease the synthetic time required for the chemical synthesis of polysaccharides, automated techniques have been developed [28–31]. Automated glycan assembly (AGA) connects monosaccharide BBs on a solid support following a linear approach (Figure 1D). Cycles of glycosylation and selective deprotection are iteratively performed to access the desired polysaccharide with full control over the length and the monosaccharide sequence [32]. Upon completion of the assembly, the desired product is released from the solid support and subjected to global deprotection. Similar to chemical polymerization, the removal of the PGs is a significant bottleneck. Additionally, several transformations remain challenging due to the poor reactivity or limited stereocontrol of certain BBs [33–37].

To overcome some limitations of the individual methods, a combination of methods is sometimes exploited. A classic example is chemoenzymatic synthesis, in which synthetic BBs are employed to direct the enzymatic synthesis towards the desired polysaccharide target.

**Figure 2:**
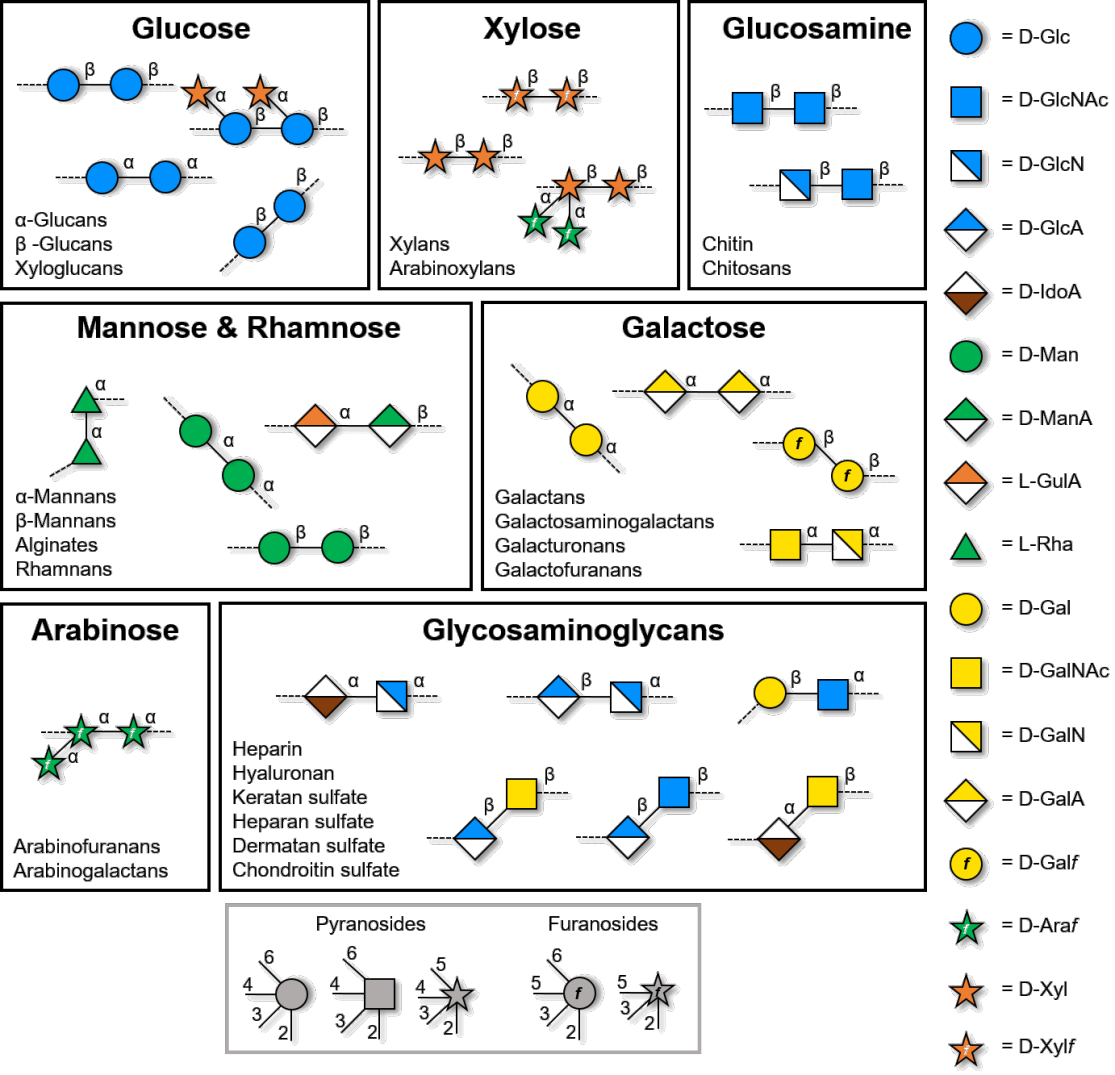
Overview of the classes of polysaccharides discussed in this review. Each section deals with polysaccharides built on the same monosaccharide backbone. Representation following the Symbol Nomenclature For Glycans (SNFG) [41].

In this review, we discuss the recent efforts towards the synthesis of well-defined polysaccharides, with particular focus on how to control the length and the substitution pattern. Each section describes a class of polysaccharides based on a particular monosaccharide backbone (Figure 2). For each class, we discuss the specific challenges associated with their synthesis and we highlight the methods that were developed to overcome such bottlenecks. We aim to provide guidelines for the synthesis of a target polysaccharide, as well as to identify the remaining bottlenecks associated with each class of polysaccharides. For this reason, the synthesis of polysaccharides based on a highly heterogeneous backbone will not be discussed [38–40]. Such syntheses are specific to the single polysaccharide target and only applicable to that particular sequence.

Each chapter focuses on recent literature describing the synthesis of oligo- and polysaccharides longer than hexasaccharides. Shorter oligomers are discussed when they represent a key step towards the synthesis of the corresponding polysaccharide. Polymers based on a sugar backbone connected via glycosidic linkages are analyzed, excluding glycopolymers and other mimetics. Extraction methodologies or post-extraction modifications are not discussed.

## Review

### Glucose-based polysaccharides

#### Cellulose

Cellulose is a polymer consisting of glucose units connected by β(1–4) glycosidic bonds. It is mainly found in two allomorphs – Cellulose I (natural) and Cellulose II (synthetic) – that differ in the orientation of the individual chains and in the number of hydrogen bonds in the crystalline structures [42–43]. In Cellulose I, the chains are oriented parallel, whereas in Cellulose II antiparallel. Natural cellulose is produced by cellulose synthases [44–46]. As the chain gets elongated, microfibrils are formed. Such fibrils are the main structural components of the plant cell-wall, together with hemicelluloses [47]. Because of the high abundance and biodegradability [48–52], cellulosic materials have found multiple industrial applications [53].

Synthesis and utilization, as well as a better understanding of cellulose’s properties are hindered by its poor solubility in most solvents. Relatively short oligomers with DPs of 6–10 tend to aggregate and precipitate out of solution [54], making isolation of pure samples troublesome. Much effort has been put to tune the synthetic conditions and produce long and uniform polysaccharides. Solvent systems like DMAc/LiCl or quaternary ammonium electrolytes are able to dissolve cellulose and could overcome the precipitation issues during synthesis [55–56]; however, these systems are not always compatible with reaction conditions [57]. The developments of synthetic cellulose until the year 2005 were discussed in a previous review [58], therefore we will focus only on recent reports. A detailed discussion on the synthesis of cellulose oligomers by enzymatic depolymerization [59] and phosphorylase [60] can be found elsewhere.

**Scheme 1:**
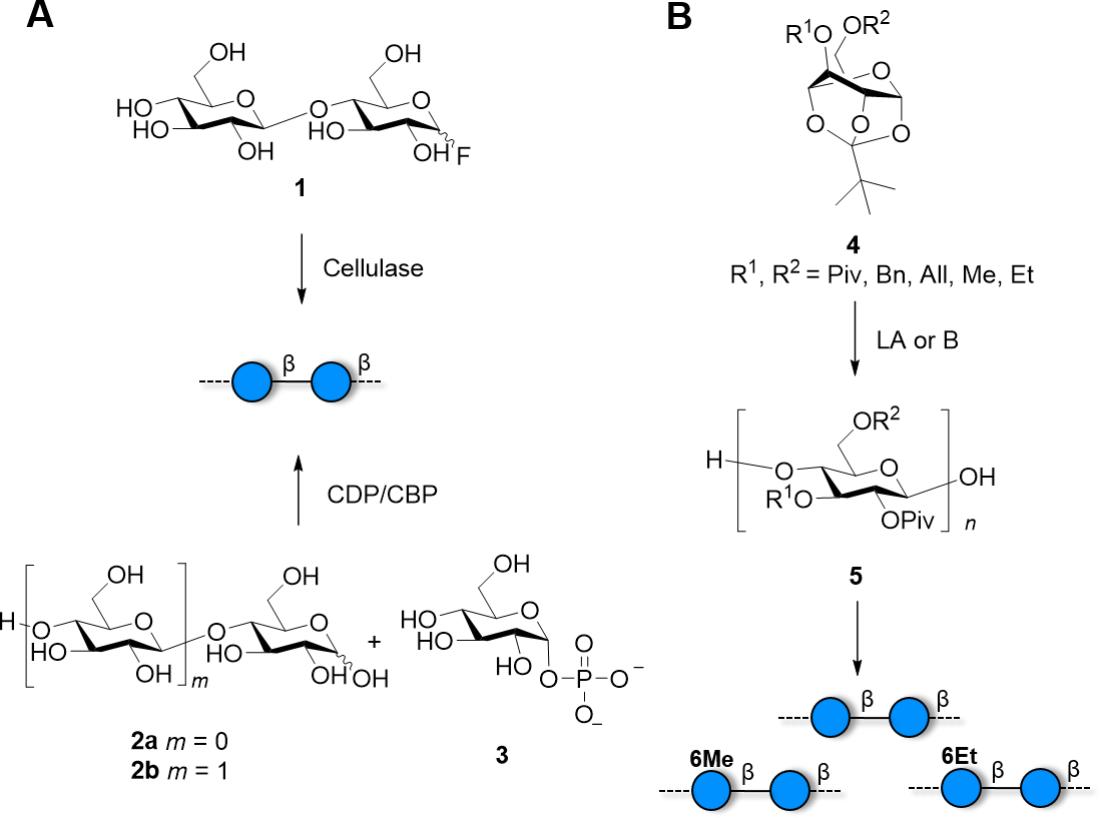
Enzymatic and chemical polymerization approaches provide cellulose oligomers with a non-uniform distribution of MW. A) Cellulose oligomers can be synthesized using cellobiose fluoride **1** and cellulase. Other enzymes like cellodextrin phosphorylase (CDP) or cellobiose phosphorylase (CBP) use glucose (**2a**) or cellobiose (**2b**) primer respectively and glucose phosphate **3**. B) Synthesis of cellulose by ROP from protected orthoester **4** promoted by Lewis acid (LA) or base (B).

The first successful enzymatic synthesis of cellulose was reported by Kobayashi in 1991 [61]. Since then, a plethora of enzymes [62] and substrates [63] were developed, aiming to narrow the dispersion of molecular weight (MW), increase the DP, or control the molecular organization of the resulting material (Scheme 1). Enzymatic polymerization of cellobiose fluoride **1** was achieved using a cellulase produced from *Trichoderma viride* (Scheme 1A). The DP of the acetylated product was shown to be at least 22. Using a purified version of this enzyme, it was possible to obtain a synthetic analogue of Cellulose I [64].

A rough control of DPs (between 5 and 14) could be obtained tuning the concentration of glucose (**2a**) or cellobiose (**2b**) primer acceptor [65]. Polymerization conducted under macromolecular crowding conditions using water-soluble polymers produced hydrogels consisting of cellulose and gelatin networks [54,66–69]. A study of the self-assembly of cellulose chains at the active site of the enzyme suggested that diffusion of the aggregated molecules and the monomers around the active center dictates the DP that can be obtained by enzymatic polymerization [70–71]. This could be the reason causing enzymatic reactions to stop after a certain chain length, as the active site becomes overcrowded. Thus, by modifying the reaction medium, a better control over the DP may be achieved.

Longer cellulose chains (DP > 100) were obtained using DMAc/LiCl as reaction solvent and a cellulose surfactant complex, even though in low yields (2–5%). This approach circumvented the precipitation of the water-insoluble oligomers and allowed for chain elongation [72–73]. This methodology was improved employing a protic co-catalyst system to yield DP > 120 in a 26% conversion [74]. Generally, an acid catalyst would promote the hydrolysis of the glycosidic bond. In this system, the SEE was hydrolytically inactive and the acid catalysis activated the C-1 at the reducing end efficiently to promote chain-elongation.

Immobilized catalysis provided cellulose analogues with very high crystallinity. A synthetic mutant enzyme of endoglucanase II [75] was cross-linked by bis-nitrilotriacetic acid yielding fibrous cellulose [76]. Cross-linked enzymes are believed to arrange their catalytic core domains and promote the formation of cellulose with high crystallinity. A similar principle was also demonstrated on a self-assembled monolayer [77]. Mimicking the natural cellulose synthases machinery can be foreseen to provide synthetic Cellulose I.

Some enzymes offer the flexibility to produce modified cellulose structures. Cellulose oligomers with fluorine substitution at C-2, C-3 or C-6 position were prepared by cellodextrin phosphorylases [78]. Mono- or multi-fluorinated compounds were obtained in good yields (30–47%) with average DPs ranging from 9 to 15. The multifluorinated compound crystallized in a new cellulose allomorph. AFM and TEM images confirmed the formation of long platelets for monofluorinated compounds, similar to those observed for cellulose [67,79], while the multifluorinated compound formed considerably shorter platelets. Cellulose–xylan [80] and cellulose–chitin [81] hybrids were also obtained upon enzymatic polymerization of the respective dimers. An amino or azido functionality in the C-6 position allows for further modifications, for example by click-chemistry [82]. These results show that enzymatic methods can be powerful approaches also to create unnatural polysaccharide materials. Nevertheless, the inherent selectivity of the enzymes limits these approaches to particular patterns and modifications.

To access a broader scope of cellulose modifications, chemical polymerization is a more suitable option. Ring-opening polymerization (ROP) of orthoesters **4** (Scheme 1B) is an established procedure for the synthesis of β(1–4)-glucopyranan structures. The first chemical synthesis of stereoregular cellulose was achieved by ROP of 3,6-dibenzyl-protected **4** catalyzed by Ph_3_CBF_4_ [83]. The protected polymer **5** was obtained in 2 h in 62% yield and an average DP of 19.3. The benzyl (Bn) group seems to be essential for the selective transformation [84]. Several derivatives were prepared in a similar fashion (Scheme 1B), including the 6-deoxy [85], ^13^C-labelled [24], ʟ-Glc [86], and ethyl/methyl analogues [87–89]. Depending on the methyl/ethyl content, it was possible to tune the solubility of the polymer in water. 6-*O*-Methyl- and 6-*O*-ethyl-celluloses were poorly soluble in water, in contrast to heterogeneous polymers with an increased content of ethyl groups. Even though the methyl/ethyl content could be adjusted using different ratios of the respective BBs, no control over the substitution pattern could be achieved. Removal of the Bn PG posed a bottleneck, requiring repeated treatments at high pressures and temperatures. Allyl groups provided a valid alternative and could be removed with palladium chloride at 60 °C in 4 hours [90].

**Scheme 2:**
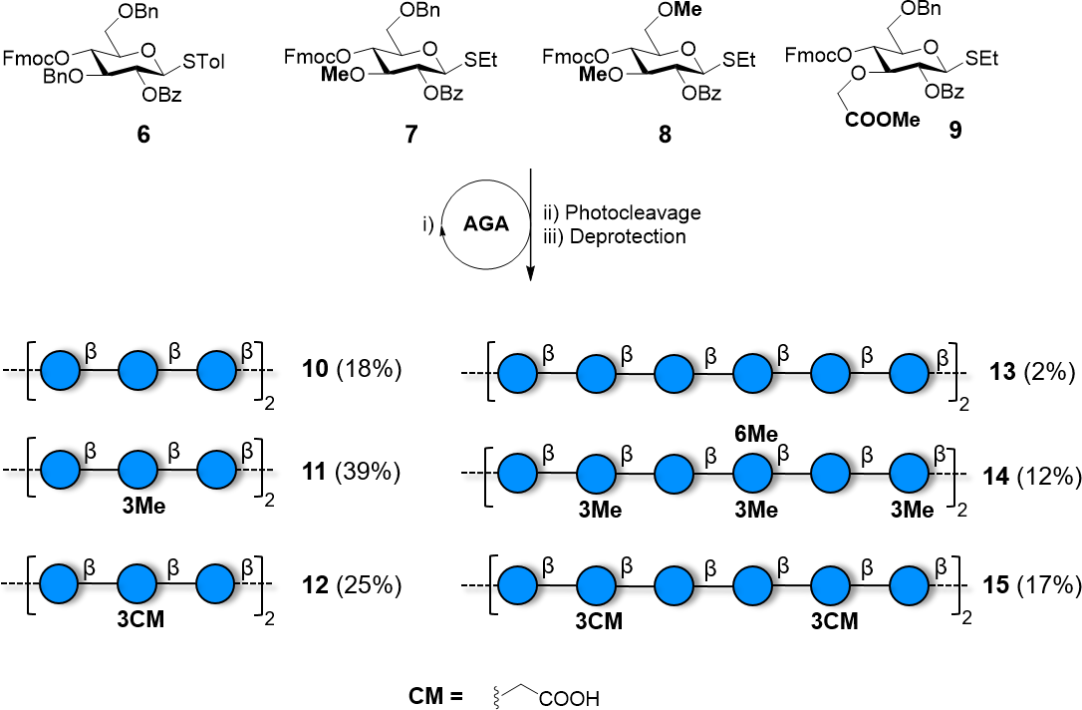
AGA of a collection of cellulose analogues obtained using BBs **6**–**9**. Specifically placed modifications prevented the formation of insoluble aggregates resulting in higher isolated yields. The AGA cycle includes coupling (NIS/TfOH), capping (Ac_2_O) and Fmoc deprotection (piperidine or Et_3_N).

Chemical synthesis grants full control over the polymer length, avoiding non-uniform dispersions of MW obtained by polymerization. Polymers with virtually any possible pattern of modification can be prepared. Strategically introduced PGs can be selectively removed to insert a branch or a chemical modification. However, to date, this approach has suffered dramatically from the severe aggregation and insolubility of cellooligosaccharides, permitting to obtain only relatively short structures in low yields. To overcome this issue, the products were converted and studied as their acetate analogues, as in the case of a cellulose 20mer obtained with a convergent approach [91–92]. To date, the longest well-defined cellulose analogue produced via chemical synthesis is a 12mer (**13**, Scheme 2), obtained in a 2% yield due to formation of insoluble aggregates during the deprotection step, resulting in loss of product during purification [93]. **13** was obtained by AGA as part of a collection of substituted compounds, obtained in much higher yields. Specifically placed substituents including methyl, fluorine, and carboxymethyl groups, prevented the formation of insoluble aggregates by disrupting hydrogen-bond networks. Dramatic differences in the conformation (e.g., radius of gyration and glycosidic bond conformation) and aggregation behaviour (i.e*.*, crystallinity and solubility) were observed for compounds with the same degree but different pattern of substitution, underscoring the importance of pure and well-defined polysaccharides for proper structure–property characterization. For example, compounds with an alternated methylation pattern resulted in quasi-linear structures, whereas more bent geometries were observed with a block wise arrangement of methyl groups. As chemical synthesis offers high flexibility in terms of manipulation, non-carbohydrate moieties can be exploited to guide the geometry of the resulting compounds. β(1–4)-Linked glucose chains were connected parallel via an anthraquinone moiety to give synthetic Cellulose I [94].

Overall, enzymatic synthesis is an established procedure to obtain non-uniform cellulose oligo- and polysaccharides. Some success was achieved using additives and molecular crowding conditions; however, this approach remains limited by product insolubility and enzyme flexibility. ROP allowed for the introduction of modifications in the structure, thus providing a first step towards structure–properties correlations. Yet, the substitution pattern remains random. Cellulose analogues were obtained with full control over the length and substitution pattern by chemical synthesis, albeit in low yields. Selective disruption of intra- and intermolecular hydrogen bonds suggested that modifications can be designed to prevent precipitation and guide cellulose assembly.

#### Xyloglucans

**Figure 3:**
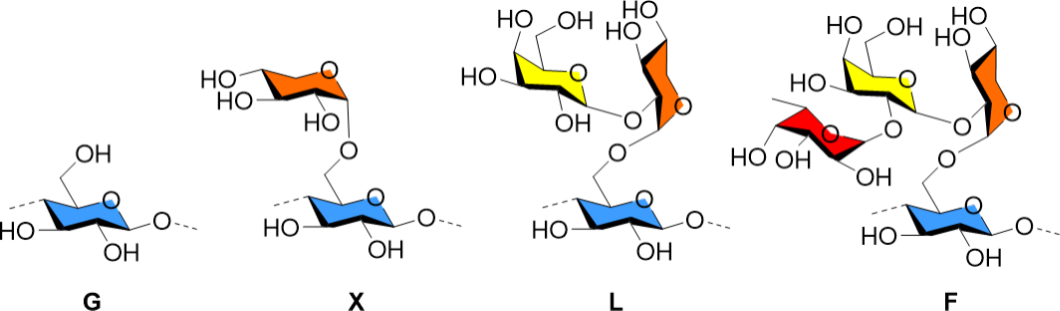
Chemical structure of the different branches G, X, L, F commonly found in XGs. Names are given following the established nomenclature [100].

Xyloglucans (XGs) are based on a β(1–4)-Glc backbone decorated with xylopyranose branches, which can be further substituted at the C-2 position by a β-linked galactose or a α-ʟ-fucose unit (Figure 3). XGs are abundant components of the plant cell-wall, where they are believed to interact with cellulose promoting the integrity of the plant wall [95]. However, a recent study suggested that XGs are not essential for the creation of the cellulose network [96].

The complexity and diversity of XGs hinder isolation from natural sources and complicates their description. A significant effort was put towards the development of enzymes for selective introduction of the side chains [97–98]. A detailed overview of the enzymatic strategies to generate artificial plant cell wall polymers was recently published [11,99]. Here, we describe the methods available to access well-defined XG oligo- and polysaccharides.

**Scheme 3:**
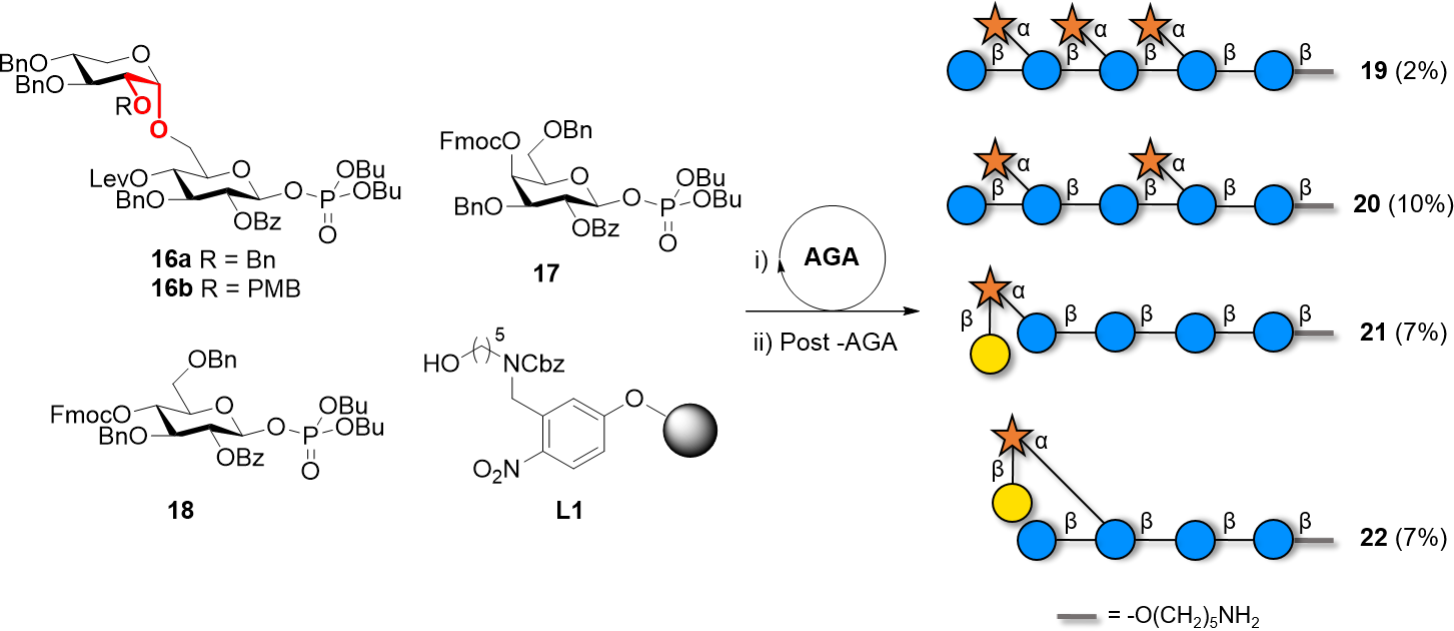
AGA of XG analogues with defined side chains. The AGA cycle includes coupling (TMSOTf), Fmoc deprotection (Et_3_N), PMB deprotection (DDQ) and Lev deprotection (hydrazine).

The first solution phase synthesis of a nonasaccharide repeating unit was realized using a convergent approach [101]. The process was later simplified using AGA and several XG oligosaccharides were prepared [102]. Representative examples are **19** and **20** (Scheme 3) [103], as well as the galactose-containing compounds **21** and **22** [104]. These compounds permitted to study XG recognition by plant cell-wall antibodies. To avoid performing the challenging 1,2-*cis* glycosylation that would generate a mixture of anomers in AGA, the α(1–6) linkage between glucose and xylose was pre-installed in the disaccharide BB **16** (Scheme 3, highlighted in red) [104]. The orthogonal levulinoyl (Lev) and *p*-methoxybenzyl (PMB) PGs could be selectively cleaved to allow for chain elongation and for introduction of the Gal unit at the C-2 of the xylopyranose, respectively.

An alternative approach utilized natural sourced XLLG tetrasaccharide, which was obtained from tamarind seed XG via enzymatic digestion. This compound was then chemically transformed to the corresponding glycosyl donors (4-methoxyphenyl- or fluoro-glycoside) to chemoenzymatically produce complex XGs. Compounds with different substitution patterns were obtained using a glycosynthase from *Humicola insolens* [105–106]. This approach produced polysaccharides with controlled substitution, but no control over the length [107].

Similarly, XG fragments prepared by enzymatic degradation of XGs were converted to the fluoride donors and used in the subsequent glycosynthase-catalyzed transformations. (XXXG)_3_, (XLLG)_3_, and XXXG-GGGG-XXXG were prepared to study interactions with bacterial microcrystalline cellulose [108]. The glycosyl fluoride donors were also polymerized to give (XXXG)*_n_* and (XLLG)_n_ with MW up to 12000 [109]. The synthesis of the highly branched (XLFG)*_n_* polysaccharides required the additional fucosyltransferase *At*FUT1 to introduce the fucose residues [110].

Even though the complexity of plant polysaccharides hinders their synthesis, these compounds are highly desirable to dissect the different interactions taking place in the plant cell-wall. Recognition of the branched oligomers by glycosynthases proved to be limited and more enzymes are needed to broaden applications and generate uniform polymers. Chemoenzymatic methods provided XG oligo- and polymers, but are laborious, requiring several synthetic steps for the synthesis of the glycosyl donors.

#### β(1–3)-Glucans

β(1–3)-Glucans, linear or with β(1–6) appendances, are present in the cell-wall of fungi and yeasts, and are major polysaccharides in brown seaweeds (laminarins) [111]. Due to their immunostimulating, antibacterial and antitumor activities, linear and branched β(1–3)-glucans have become interesting synthetic targets. Since several reports highlighted the correlation between chain length and biological activity, significant effort has been put to chemically synthesize β(1–3)-glucans with well-defined lengths.

**Figure 4:**
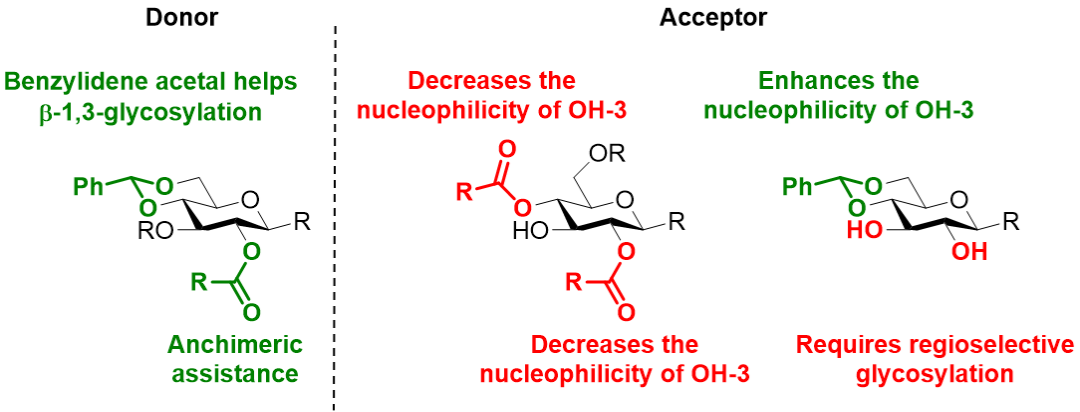
Synthetic strategies and issues associated to the formation of the β(1–3) linkage.

In contrast to β(1–4)-glucans (i.e., cellulose), β(1–3)-glucans with a DP up to 20 can be dissolved in water [112], simplifying the synthetic process. Several oligosaccharides have been prepared to identify the best PG pattern ensuring a proper balance between BB reactivity, stereoselectivity, and simplicity of deprotection [113–125]. Ester groups are commonly employed at C-2 position to ensure anchimeric assistance during glycosylation (Figure 4). However, the *O*-2 acyl group on the glycosyl acceptor hinders the 3-*O*-glycosylation sterically and electronically [126]. The decreased nucleophilicity of the C-3 hydroxy acceptor could lead to poor stereoselectivity [127]. This issue becomes even more dramatic when 2,4-di-*O*-acyl groups are present, sometimes leading to exclusive formation of α-anomers [128–129]. PGs like 2-*O*-ADMB (4-acetoxy-2,2-dimethylbutanoate) [130] or 2,2’-*O*-benzylidene [131] were introduced to solve this issue. Several reports highlighted the importance of the 4,6-*O*-benzylidene group on the glycosyl donor and the acceptor for the stereoselective formation of the β(1–3) linkage (Figure 4) [132].

Interestingly, oligomers bearing several 4,6-*O*-benzylidene groups show anomalously small coupling constants for some of the C-1 hydrogens. NMR [133] and X-ray [134] studies revealed that, for some residues, the 4,6-*O*-benzylidene group stabilizes a boat conformation (^1,4^*B* or *B*_2,5_), in contrast to the standard chair (^4^*C*_1_). This unexpected conformation did not affect the stereoselectivity of the glycosylation, permitting the preparation of linear β(1–3)-glucans of different lengths (6mer, 8mer, 10mer and 12mer) following a convergent preactivation-based iterative strategy [135]. These compounds were subsequently conjugated to keyhole limpet hemocyanin, revealing that the length of the glucans affected the immunogenic properties. A pre-activation-based iterative one-pot glycosylation method was also employed to access different branched structures having β(1–3)- and β(1–6)-linked Glc appendances on a β(1–3) backbone [136].

**Scheme 4:**
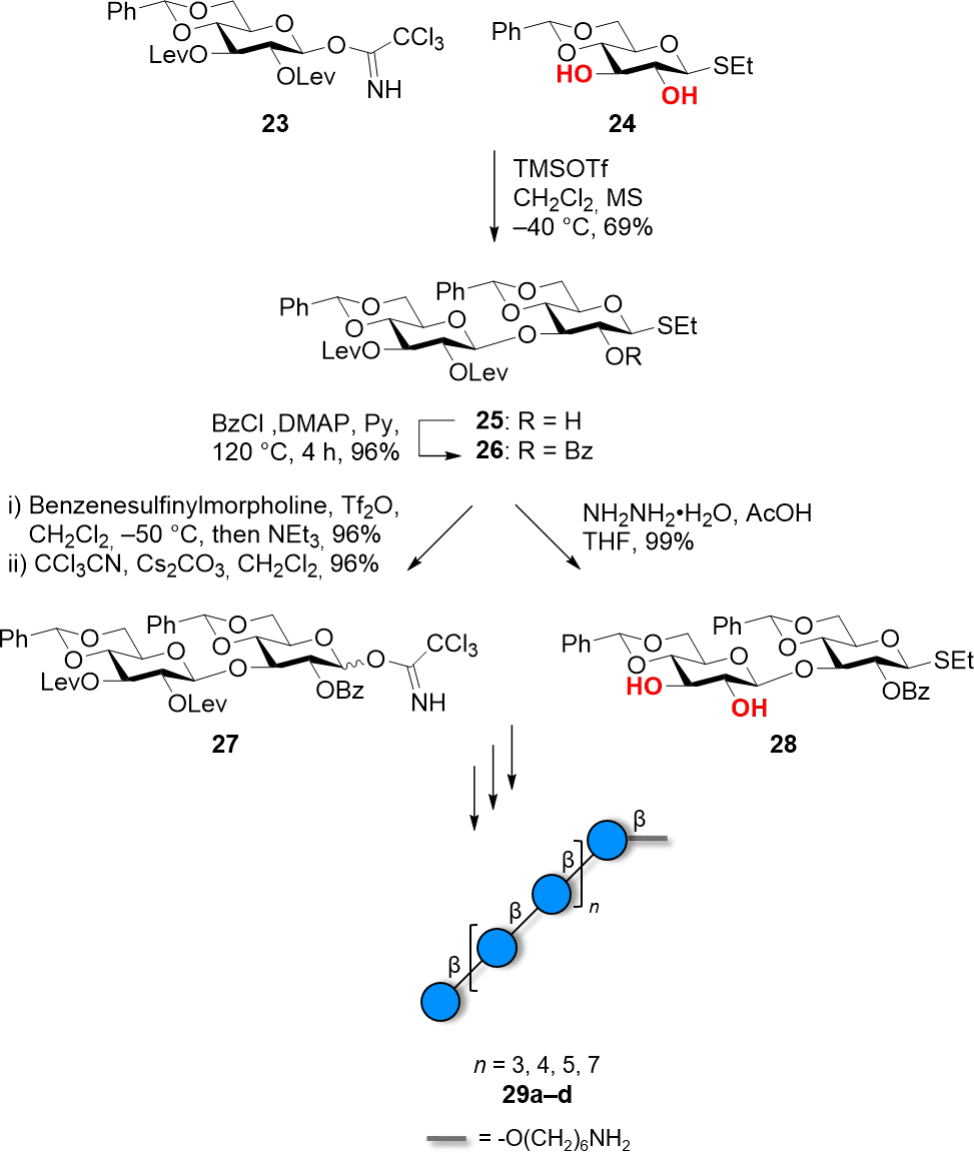
Convergent synthesis of β(1–3)-glucans using a regioselective glycosylation strategy.

To date, the longer β(1–3)-glucans [137–138] were obtained using a 2-*O*-acylated donor, to ensure β-selectivity, followed by deacylation and use of the resulting 2,3-diol as acceptor in the following glycosylation [131]. A regioselective glycosylation strategy was developed to obtain a collection of linear glucans (8mer to 16mer) (Scheme 4), as well as a branched 9mer and 17mer [137–138]. A similar strategy based on the regioselective glycosylation of a 2,3-diol 4,6-*O*-benzylidene acceptor permitted to access various β(1–3)-glucans, ranging from trimer to 13mer [126]. Regioselectivity was also achieved using a gold-catalyzed glycosylation, to give a linear β(1–3)-linked 11mer and a branched 14mer in a convergent manner [139].

Collection of linear as well as branched β(1–3)-glucans with defined lengths could be prepared by AGA [140–142]. The poor nucleophilicity of the C-3 hydroxy group on the glycosyl acceptor required the use of more reactive glycosyl phosphate donors and, in some cases, a double glycosylation cycle [118,143].

Longer structures, albeit with no control over the DP, could be prepared via chemoenzymatic polymerization of α-laminaribiosyl fluoride donors catalyzed by the mutated barley β(1–3)-glucanase [144–145]. Linear β(1–3)-glucans with DP of 30–40 appeared as lamellar, hexagonal crystals. Electron and X-ray diffraction studies revealed that long β(1–3)-glucan structures adopt a parallel, triple helical structures [146]. A glycosynthase derived from *Bacillus licheniformis* could polymerize glycosyl fluoride donors to prepare artificial mixed linkage β-glucans with an average molecular mass of 10–15 kDa [147].

The better solubility of β(1–3)-glucans, compared to the β(1–4) analogues, has permitted the chemical or enzymatic synthesis of long polysaccharides. Still, the poor nucleophilicity of the hydroxy group at C-3, in the presence of a C-2 ester PG, could decrease the efficiency of chemical synthesis. Hence, there is scope for new strategies to provide anchimeric assistance, without affecting the nucleophilicity of the hydroxy group at C-3.

#### β(1–6)-Glucans

The formation of the β(1–6)-glycosidic bond poses less synthetic challenges than the β(1–3) bond. The primary C-6 hydroxy group is more nucleophilic than the secondary hydroxy group at C-3 and β-selectivity is easily achieved with the help of anchimeric assistance provided by a 2-*O*-acyl functionality. Despite these advantages, the synthesis of β(1–6)-Glc polysaccharides appears only in few reports [148–149], with short oligomers being prepared mainly to prove new methodologies [31,150–155].

The synthesis of structures longer than hexasaccharides suffered from poor solubility during the deprotection step, likely due to the formation of aggregates resulting from particular secondary structures adopted by the partially protected intermediates [156]. The replacement of a single Glc unit with a Man unit was sufficient to disrupt this secondary structure and significantly increased the isolated yield of the final compounds. Molecular dynamic (MD) simulations predicted a compact helical conformation for the fully deprotected compound, confirmed by NMR studies on a collection of ^13^C-labelled hexamers [157]. Similar issues during deprotections were not observed when the β(1–6) backbone was substituted with β(1–3) branches [158–166] and long oligomers could be synthesized on solid support [157,166] or in solution phase using block coupling [165].

#### α-Glucans

In contrast to the relatively simple formation of a 1,2-*trans* glycosidic bond using participating group at C-2, the construction of 1,2-*cis* glycosidic bonds is a long-standing challenge in the field of glycochemistry [167]. The formation of 1,2-*cis* Glc linkages is particularly relevant, as α(1–4)-glucans form the backbone of starch, with the linear amylose and the branched amylopectin, and α(1–3)-glucans are related to fungal pathogenicity in plants [168]. Only few options can aid the stereoselective construction of 1,2-*cis* glycosidic bonds. In general, stereocontrol can be obtained either by fine tuning of PGs at C-3, C-4 or C-6 positions, or by making use of participating solvents [37,167]. 3-*O*-acyl and 6-*O*-acyl groups are commonly employed to remotely participate in the glycosylation and promote α-selectivity [169–170]. This strategy permitted to prepare oligosaccharides having multiple α-glycosidic linkages by AGA [171].

Additional strategies include the dehydrative glycosylation of glucosyl hemiacetal [172] or the halide-catalyzed in situ anomerization of glucosyl iodide donors that yielded α(1–6)-hexaglucans [173]. The halide-catalyzed in situ anomerization approach was efficient in solid phase as well as in solution phase synthesis. The participating (*S*)-(phenylthiomethyl) benzyl chiral auxiliary at the C-2 position of the glucosyl donor permitted the solid phase synthesis of a branched pentaglucan having a α(1–3) branch on an α(1–6) backbone [174]. Boron-mediated aglycon delivery (BMAD) with the use of a diboron catalyst allowed for regio-, as well as stereoselective glycosylation to achieve an α(1-3)-pentaglucoside [175]. Regioselective 1,2-*cis*-glycosylation could also be achieved by boron-catalyzed coupling via a S_N_i-type mechanism [176]. Hydrogen bond-mediated aglycon delivery (HAD) with the aid of a 4-*O*-picoloyl (Pico) group offered an interesting method to achieve multiple α-glucosidic linkages. However, the efficacy of the HAD diminished with the increased bulk of the glycosyl acceptor [177].

**Scheme 5:**
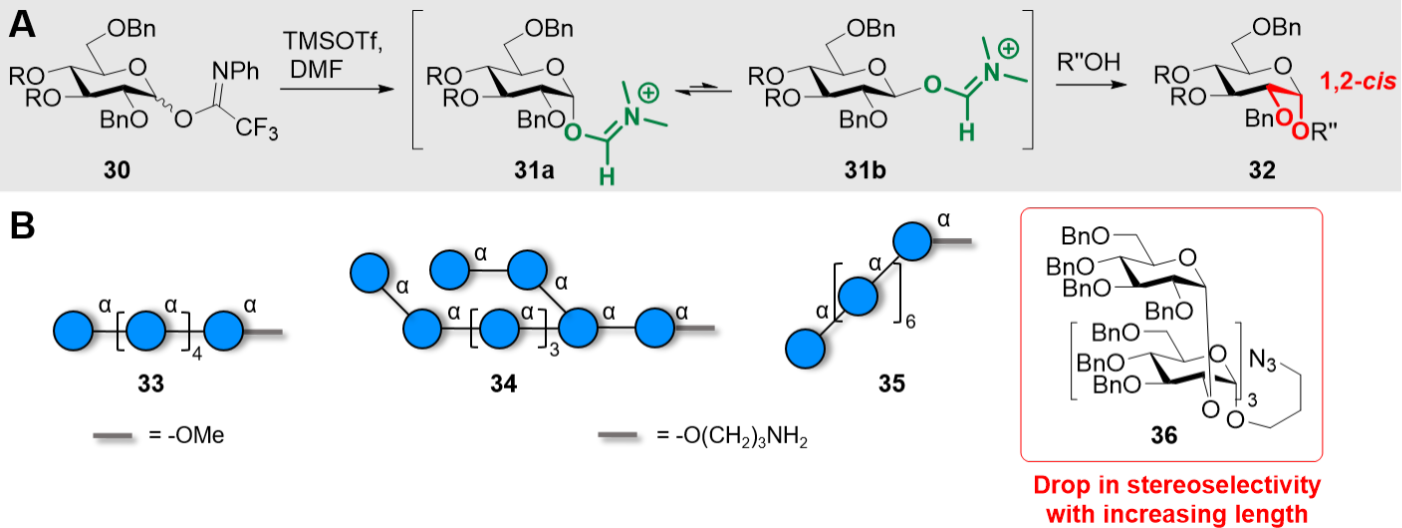
DMF-mediated 1,2-*cis* glycosylation. A) General mechanism and B) examples of α-glucans prepared using this approach.

In general, even though several elegant approaches were reported, the construction of multiple α-linkages remains challenging, in particular with the increasing size of the acceptor. The nucleophilic additive glycosylation-based approach (Scheme 5A) offers the opportunity to install multiple 1,2-*cis* glycosidic bonds by converting the glycosyl donor **30** into a less reactive adduct **31**. DMF-mediated glycosylations permitted access to long structures based on multiple α(1–4) linkages **33** and **34** [178] and α(1–3) linkages **35** [179] (Scheme 5B). The short oligosaccharide **36** having α(1–2) linkages was also prepared, however, in this case, the stereoselectivity diminished with the increasing length of the oligosaccharide, possibly due to steric hindrance [180].

**Scheme 6:**
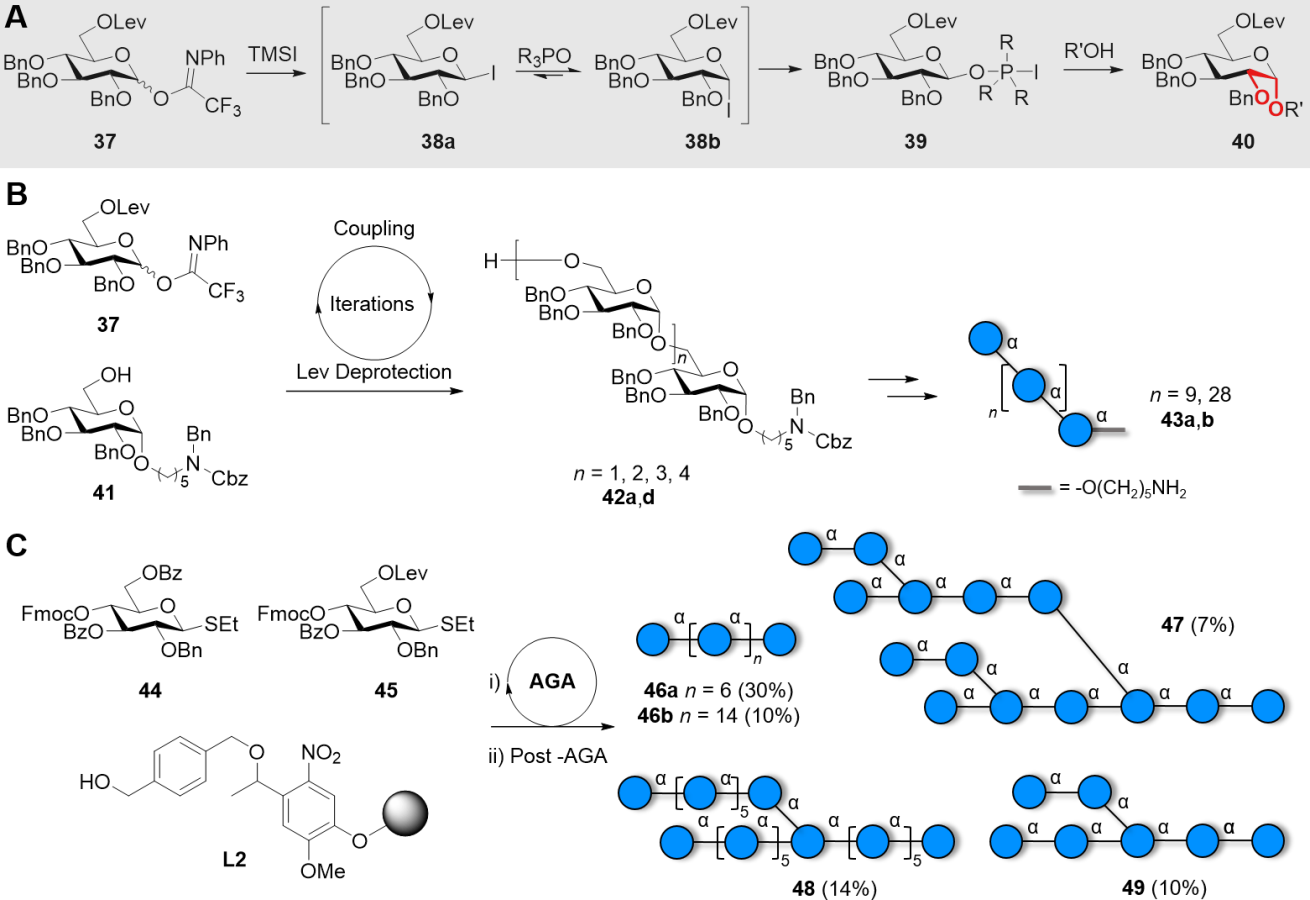
Synergistic glycosylation strategy employing a nucleophilic modulation strategy (TMSI and Ph_3_PO) in combination with the *O*-6-Lev remote anchimeric assistance. A) General glycosylation mechanism. B) Synthesis of linear α(1–6)-glucan 11mer and 30mer. C) AGA of starch and glycogen oligo- and polysaccharides. The AGA cycle includes coupling (NIS/TfOH), capping (Ac_2_O), Fmoc deprotection (Et_3_N), and Lev deprotection (hydrazine).

The nucleophilic modulation strategy in combination with the *O*-6-Lev remote anchimeric assistance could further improve the stereoselectivity of the 1,2-*cis* glycosylations, leading to the synthesis of long linear α-glucans **43** (11mer and 30mer) having multiple 1,2-*cis* glycosidic linkages (Scheme 6A and 6B) [181]. The remote anchimeric assistance from ester groups at C-3 and C-6 on thioglucoside BBs **44** and **45** permitted the AGA of linear **46a**,**b** and branched **47–49** glucans with multiple 1,2-*cis* linkages (Scheme 6C) [182].

To avoid the chemical formation of this challenging bond, natural compounds containing 1,2-*cis* linkages could be exploited. α-, β- and γ-cyclodextrins offered a good starting material to prepare stereoselectively a 6-*O*-methylated α(1–4)-glucopolysaccharides consisting of 6–20 monomer units [183–184]. As alternative, stereoselectivity can be achieved with enzymes. Glucan phosphorylase is commonly employed to produce linear amylose polysaccharides with the desired average MW by changing the glucose monophosphate/amylose primer ratio [185]. This approach permitted the preparation of amylose with narrow MW distribution, amylose hybrids, and amylose-functionalized materials [186]. Linear and branched oligosaccharides with mixed α(1–3) and α(1–4) linkages were synthesized using a glucansucrase from *Lactobacillus reuteri* [187–188].

Even though several chemical methods are available for the formation of 1,2-*cis* linkages, only few are unaffected by the increasing size of the target molecule. The recently published synergistic approach that combines remote anchimeric assistance and nucleophilic modulation permitted access to long linear α-glucans [181]. The implementation of a similar strategy in AGA has already shown promising results [182] and could fuel the production of well-defined polysaccharides based on multiple 1,2-*cis*-glycosidic linkages.

### Xylose-based polysaccharides

Xylans are abundant polysaccharides mainly found in plants or in mammalian cells as proteoglycans [189–190]. They participate in the formation of the cell-wall by interacting with cellulose microfibrils [191], however, these interactions lack a molecular description [192]. Some evidences suggest that the xylan–cellulose interaction depends on the xylan substitution pattern [193].

Natural xylans contain β(1–4), β(1–3) or a combination of β(1–4)- and β(1–3)-linked β-ᴅ-xylopyranosyl units. Rarely, α(1–3) glycosidic linkages are present [194]. These backbones are often partially acetylated or appended with sugar side chains, mainly ʟ-arabinose, fucose, galactose and 4-*O*-methylglucuronic acid [195]. In general, low substituted xylans are forming insoluble aggregates, whereas heavily substituted xylans are highly soluble in water [196–197]. Minor changes to the xylan structure can dramatically affect their crystallinity and solubility [198], highlighting the importance of obtaining well-defined samples.

**Scheme 7:**
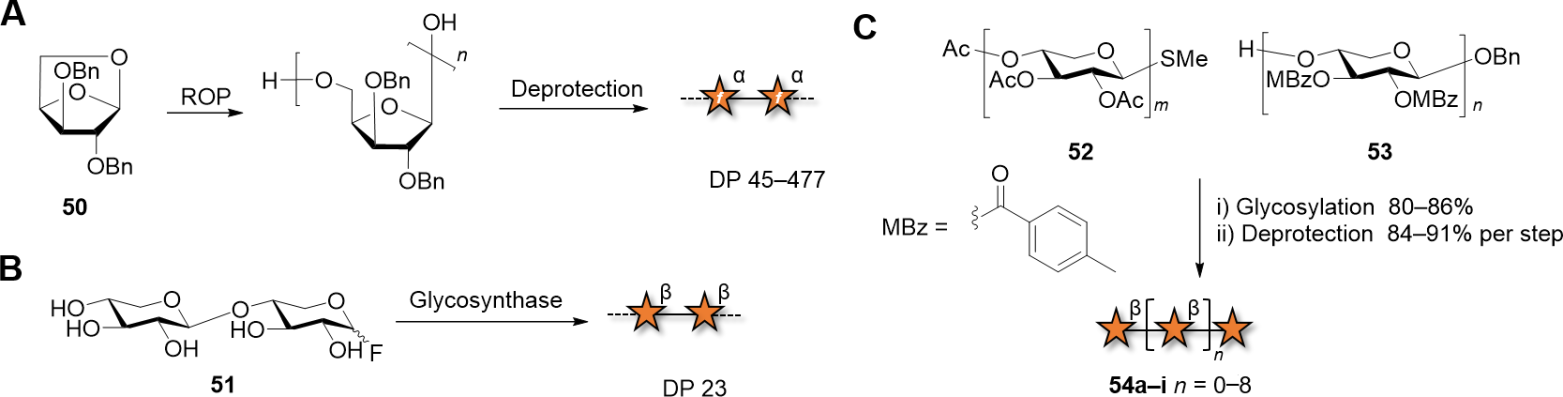
Different approaches to produce xylans. A) Polymerization techniques including ROP, and B) enzymatic polymerization yielded non-uniform homopolymers. C) Chemical synthesis following a convergent approach provided short oligomers with control over DP.

Cationic ROP of anhydrosugars was the pioneering approach for the preparation of synthetic, unnatural xylans. Polyxylofurans with (1–5) and (3–5) linkages were described [199–200]. In most cases, non-uniform polysaccharides were obtained, as in the case of α(1–5)-xylans, prepared in up to 93% yields and DP between 45 to 477 (Scheme 7A) [201]. Enzymatic polymerization is generally performed using glycosyl fluorides as substrates, which are structurally related to the natural glycosyl enzyme intermediate [202]. β-Xylobiosyl fluoride donors, selectively recognized by the cellulase from *Trichoderma viride,* afforded xylans containing exclusively β(1–4) linkages in 72% yield [203]*.* Compounds with DP higher than 23 constituted the insoluble fraction (37% yield) (Scheme 7B). The combination of XynB2-catalyzed dimerization of α-ᴅ-xylopyranosyl fluoride and subsequent polymerization by XynA helped to overcome the bottleneck of poor substrate-recognition of longer oligosaccharides by glycosynthases [204]. Still, the polymer was obtained with a wide range of DP between 6–100. Shorter Xyl chains with up to 12 units were reported using a retaining xylanase from *Cellulomonas fimi* [202].

Well-defined xylan oligosaccharides were obtained by chemical synthesis following convergent or sequential glycosylation approaches. These well-defined xylans allowed establishing correlations between the substitution pattern, length, and macroscopic properties (i.e., crystallinity, solubility, interactions with other polysaccharides). To date, the longest xylans reported are 10mers [205], although there is in principle no reason hindering the synthesis of longer structures. A convergent approach is often employed to avoid tedious purification of unreacted acceptors from the products, resulting from incomplete glycosylation. Peracetylated thioglycoside donors **52** were reacted with 4-methylbenzoylated (MBz) acceptors **53**, following NIS/AgOTf activation (Scheme 7C). Upon global deprotection, a collection of well-defined oligoxylans with 4–10 monosaccharide units was obtained [205]. A convergent approach was also employed to prepare a β(1–3)-Xyl hexamer, an analogue of xylans found in algae cell-walls [206]. TMSOTf promoted the glycosylation of the Bz-protected disaccharide acceptor with the Bz-protected tetrasaccharide trichloroacetimidate donor. Although not yet reported, the authors suggested that this method can be extended to the synthesis of longer oligomers in a gram-scale.

**Scheme 8:**
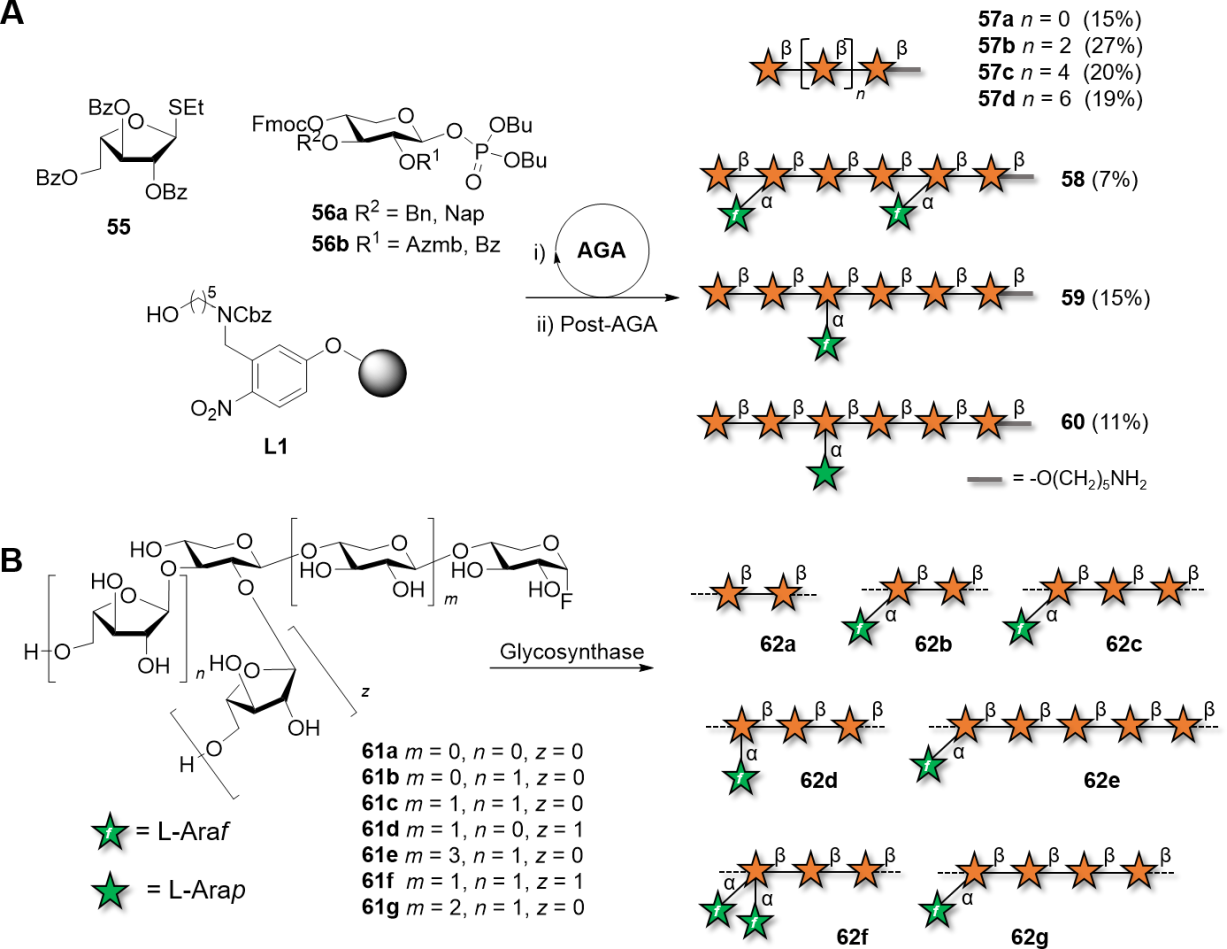
A) Synthesis of arabinofuranosyl-decorated xylan oligosaccharides using AGA. Representative compounds are reported. The AGA cycle includes coupling (NIS/TfOH for thioglycosides and TMSOTf for glycosyl phosphates), Fmoc deprotection (Et_3_N) and Nap deprotection (DDQ). Azmb = 2-(azidomethyl)benzoyl. B) Chemoenzymatic synthesis of arabinofuranosyl-decorated xylan polymers with well-defined substitution patterns.

To avoid purifications after each glycosylation step, oligoxylans were produced by AGA on solid support (Scheme 8A) [207–208]. The iterative addition of monosaccharide BBs and the use of orthogonal PGs permitted to control the length and pattern of substitution. A collection of linear β(1–4)-ᴅ-xylan chains and with α(1–3)-ʟ-arabinofuranosyl branches was quickly assembled to identify the binding epitopes of monoclonal antibodies. These oligomers, together with other well-defined analogues obtained by chemoenzymatic synthesis, were converted to the respective glycosyl fluoride and used for enzymatic polymerization catalyzed by XynAE265G (Scheme 8B) [7]. This approach generated polymers **62a–g** with well-defined substitution patterns. Differences in solubility were observed for certain patterns, affecting the efficiency of polymerization and the DPs of the resulting polymers. Linear xylan precipitated with a MW of 9.2 kDa. Arabinofuranosyl xylans substituted at the C-2 or C-3 position of every third residue, formed crystalline aggregates. The authors speculated that a threefold helical screw conformation might enable the regular interaction of individual chains. The arabinose pattern also played a role in the interaction with cellulose, with the crystalline compound **62c** that did not adsorb to cellulose, in contrast to **62b**, which adsorbed irreversibly. The high adsorption of **62b** was associated with its twofold helical screw conformation, only accessible with this substitution pattern.

Overall, chemical and enzymatic polymerization are faster than chemical synthesis, however, non-uniform xylans are always generated. The chemoenzymatic approach permitted to prepare, for the first time, arabinose-substituted polymers with a defined substitution pattern. Although still lacking control over the polymer length, these compounds underscored the importance of well-defined polysaccharides to understand the microscopic properties of natural xylans and fuel advancements in plant cell-wall biology [102].

### Glucosamine-based polysaccharides

#### Chitin and chitosan

Chitin is a linear polysaccharide composed of β(1–4)-linked 2-acetamido-2-deoxy-ᴅ-glucopyranose (GlcNAc) repeating units, which mainly exists in the exoskeleton of crustaceans and insects, as well as in the cell-wall of fungi [209–210]. Chitosan, its partially *N*-deacetylated analogue, has vast industrial applications in coating materials, cosmetics and pharmaceuticals [211]. Owing to their superior biological and mechanical properties, chitin and chitosan are used in fibers, gels, sponges, films, beads, and nanoparticles [212]. Tunable stiffness, solubility, and transparency can be obtained by tuning of the DP and fraction of acetylation (FA).

Chitooligosaccharides (COS: β(1–4)-linked oligomers of GlcNAc and/or GlcN) have gained popularity due to their exceptional antimicrobial, antitumor, and immune modulatory activities [29,213–217]. Methods to obtain well-defined COS with controlled size and substitution pattern are highly desirable, because DP and FA affect the physicochemical properties of the COS [218–219]. A size-dependent immune recognition was verified in plant chitin receptors as well as in toll-like receptors (TLR2) [220]. Moreover, the PA can tune the biological activity, as demonstrated using partially acetylated COS obtained by enzymatic degradation of chitosan promoted by hydrolases [221].

Chemical degradation of chitin promoted by acid treatment [222–228] or using chitinases and chitosanases [229–231] generates mixtures of short COS, which requires tedious purification steps. Furthermore, degradation of natural chitin often lacks proper control over the pattern of acetylation (PA). Acetolysis also often leads to heterogeneous mixtures [232–235]. A controlled acetolysis of chitin, followed by the one-pot *trans-N*-trifluoroacetylation of peracetylated chitooligomers, could efficiently generate 1,2-(2-trifluoromethyloxazoline) donors. These reactive compounds were employed to construct different length COS, from dimer to hexamer [236].

**Scheme 9:**
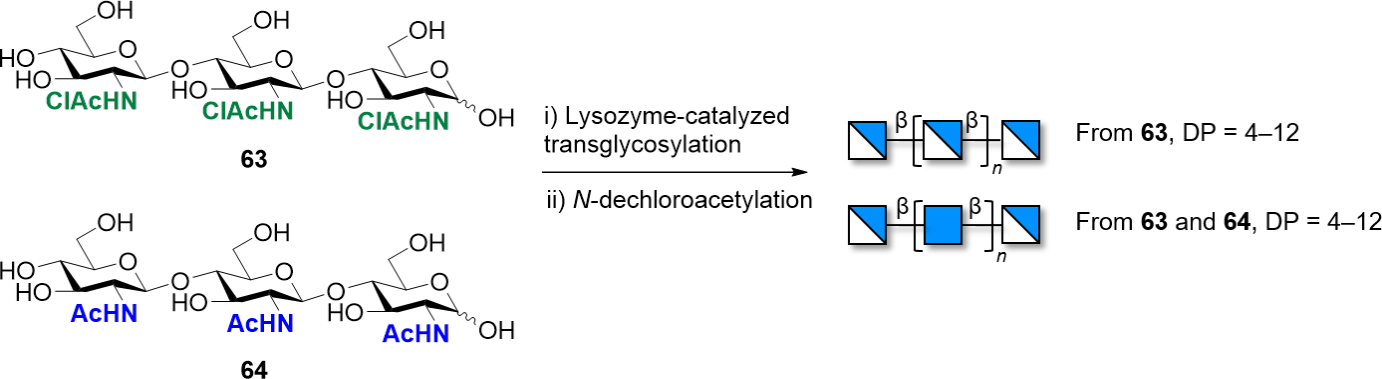
Chemoenzymatic synthesis of COS utilizing a lysozyme-catalyzed transglycosylation reaction followed by the deprotection of *N*-chloroacetate.

Synthetic COS were prepared following chemical or enzymatic polymerization, albeit with poor control over size and substitution pattern. Classical methods include chitinase-catalyzed assembly via ring-opening polyaddition of *N,N’*-diacetylchitobiose oxazoline derivatives [237–239] or self-condensation of *N*-phthalimide protected thioglycoside [240]. Enzymatic polymerization promoted by hydrolases is an interesting option to produce long COS from shorter oligomers [241]. After cleaving the glycosidic linkage, the enzyme remains attached to the new reducing end of the oligomer, releasing the cleaved part. Subsequently, the oligomer is transferred to another COS acceptor, forming a new glycosidic bond [216]. Various enzymes have been successfully employed, including β-*N*-acetyl-hexosaminidase [242], lysozyme (Scheme 9) [243], and endochitinase [244–246]. With these approaches, structures with a DP up to 13 could be accessed. Genetically engineered transglycosylating hydrolases were employed to polymerize chemically activated oligosaccharide substrates [216]. These chemoenzymatic approaches are often based on glycosynthase-type GH18 or GH19 chitinases and produce mixture of COS with different lengths [237,243,247–250].

**Scheme 10:**
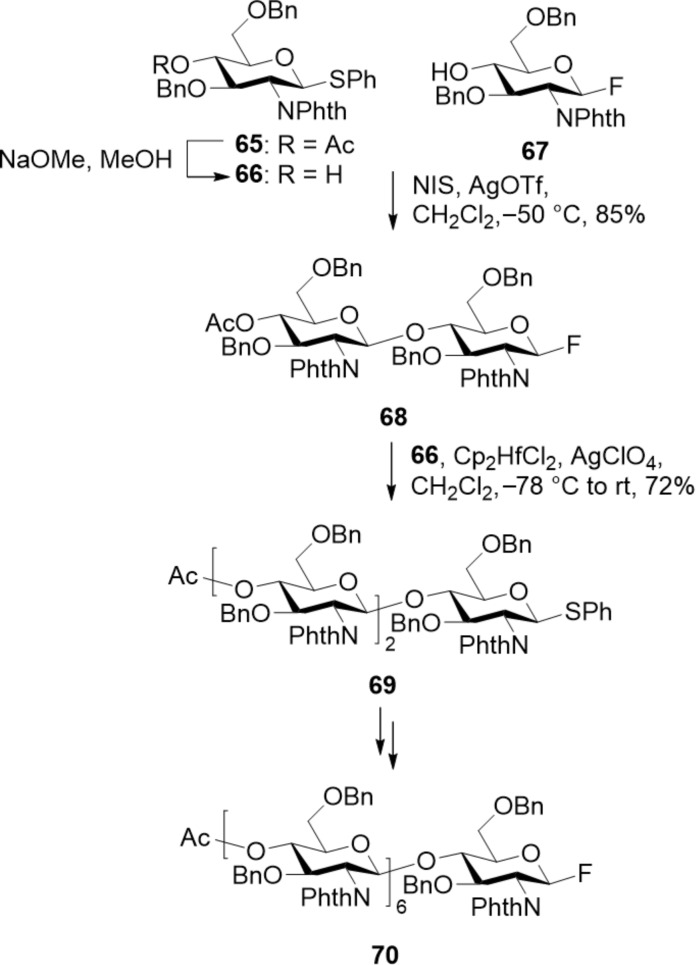
Synthesis of COS using an orthogonal glycosylation strategy based on the use of two different LGs.

Chemical synthesis offers the possibility to generate well-defined COS, but it is to date underexploited. The poor reactivity of the C-4 hydroxy group of the glucosamine BB, and the need of orthogonal PGs on the nitrogen atom to control the PA are the main bottlenecks [251]. The β-directing *N*-phthaloyl (*N*-Phth) PG was installed in trichloroacetimidate donors to synthesize fully deacetylated COS up to 12mer. After each glycosylation step, the 4-methoxyphenyl group at the anomeric position was oxidatively removed and the resulting hemiacetal was transformed into the trichloroacetimidate glycosyl donor for the next glycosylation step [252]. An orthogonal glycosylation strategy was developed to alternately stitch *N*-Phth-protected thioglycoside donors and *N*-Phth-protected fluoride donors to obtain COS up to 7mer (Scheme 10) [253]. Similarly, trichloroacetimidate and thioglycoside donors permitted the synthesis of short COS [254]. Upon assembly, the *N*-Phth PGs were removed with hydrazine under reflux and the free amino groups were acetylated to obtain the fully *N*-acetylated COS [255].

**Scheme 11:**
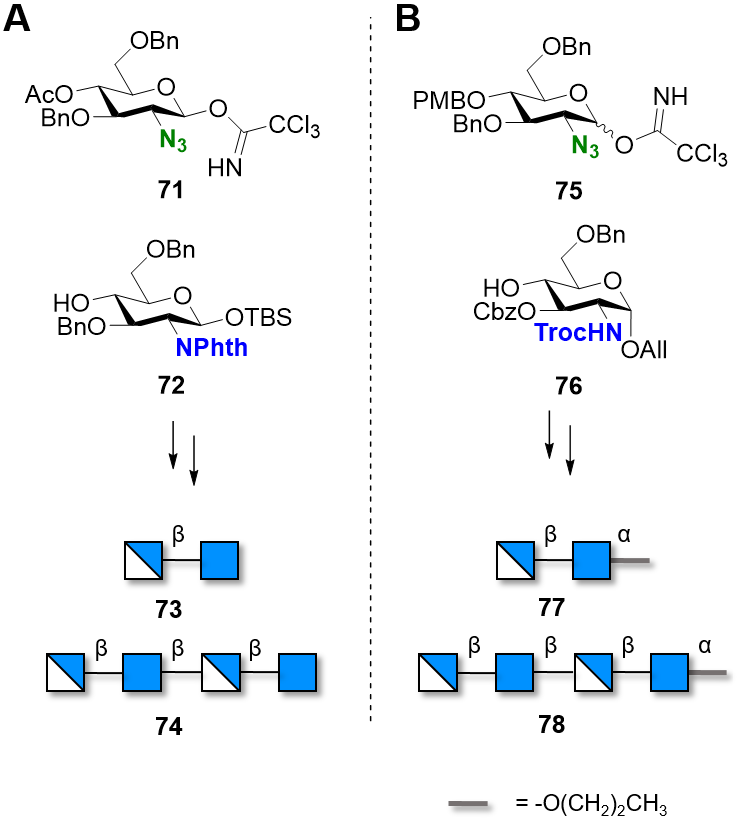
Orthogonal *N*-PGs permitted the synthesis of COS with different PA.

Control over the pattern of *N*-acetylation was for the first time achieved using two monosaccharides bearing an azido (N_3_) and a *N*-Phth moieties as precursors of the free and *N*-acetylated amino group, respectively [256]. The stereoselectivity of the glycosylation reaction between the C-2 azido donor **71** and *N*-Phth **72** acceptor met with little success, lowering the yield of the desired anomer significantly. The obtained disaccharide was converted to the trichloroacetimidate donor and used in a [2 + 2] glycosylation, affording the tetramer **74** with an alternated *N*-acetyl pattern (Scheme 11A) [257]. Partially *N*-acetylated COS dimer and tetramer were obtained exploiting the orthogonality of the azide and *N*-Troc groups [258]. β-Selectivity during the glycosylation with the C-2 azido trichloroacetimidate donor **75** was controlled by S_N_2 displacement of the α-trichloroacetimidate LG upon activation with BF_3_·OEt_2_ in CH_2_Cl_2_/*n*-hexane (3:2) (Scheme 11B). The orthogonal *N*-trichloroacetyl (*N*-TCA) and *N*-benzyloxycarbonyl (*N*-Cbz) PGs permitted the synthesis of chitobioses with different PA. *N*-TCA groups could be converted into *N*-acetates upon reduction with tributyltin hydride and azobisisobutyronitrile (AIBN), and the *N*-Cbz groups were removed to liberate the free amino groups during hydrogenolysis over Pd/C [259].

**Scheme 12:**
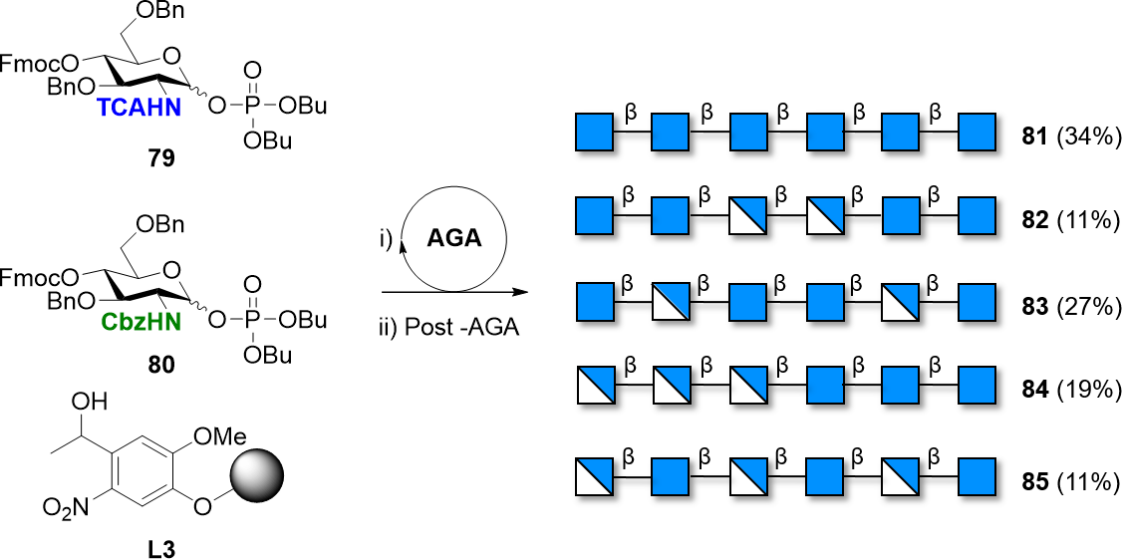
AGA of well-defined COS with different PA using two orthogonally protected BBs. The AGA cycle includes coupling (TMSOTf), capping (Ac_2_O), and Fmoc deprotection (piperidine).

A collection of well-defined COS with defined DP and PA was prepared by AGA [93,157,260]. The automated solid-phase approach required only two BBs with the amino group protected either with the *N*-TCA **79** or with the *N*-Cbz group **80** (Scheme 12). Compounds **81**–**85** served as standards to explore the conformational space of COS with different PA. NMR analysis and MD simulations revealed the importance of the deacetylated residues, with the free amino group able to stabilize new geometries.

Many more *N*-protecting groups [217,261] are available and could generate COS with defined PA, however, to date most of them have shown significant drawbacks, decreasing the reactivity of the BB during glycosylation or complicating the deprotection steps. Orthogonality and stability become particularly crucial when the goal is the multistep synthesis of long COS.

#### Other glucosamine-based polysaccharides

**Scheme 13:**
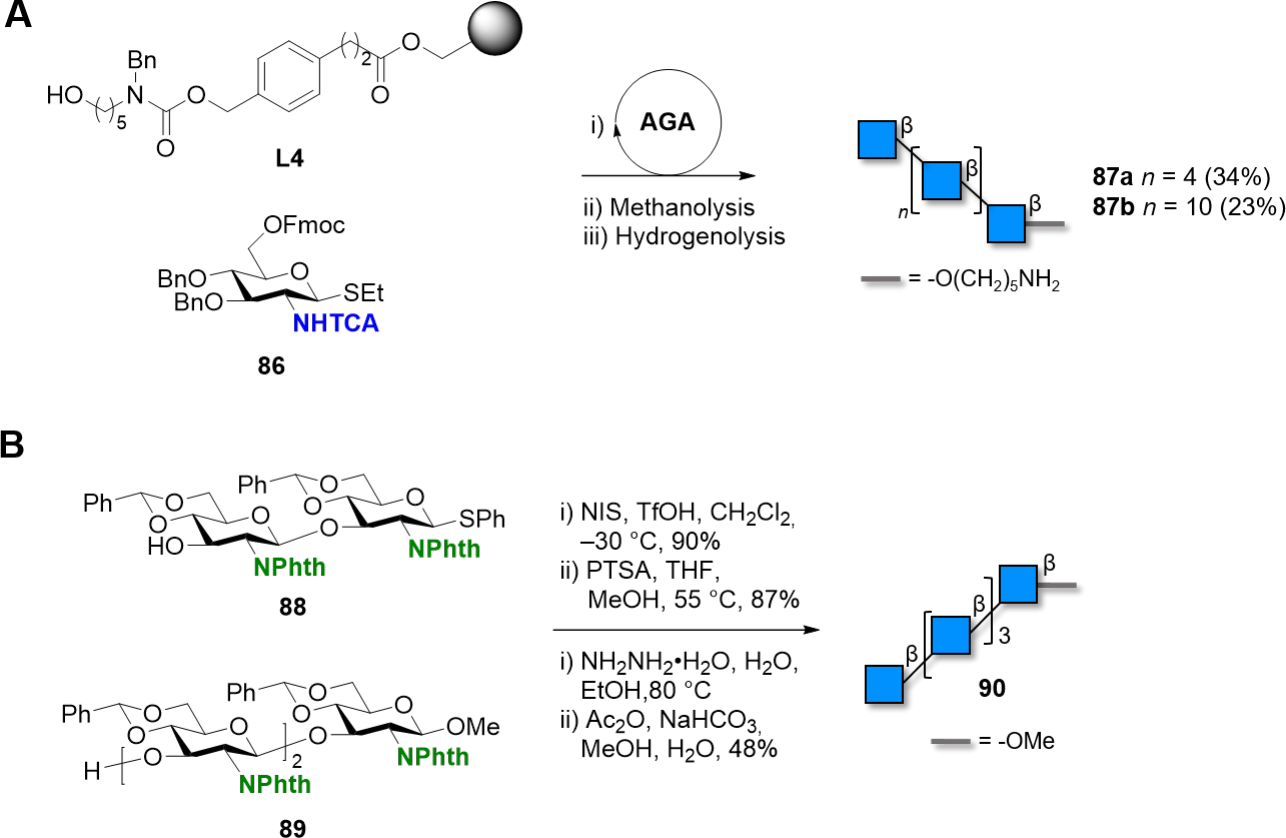
A) AGA of β(1–6)-*N*-acetylglucosamine hexasaccharide and dodecasaccharide. AGA includes cycles of coupling (NIS/TfOH) and Fmoc deprotection (piperidine). B) Convergent synthesis of a β(1–3)-glucosamine pentasaccharide.

Other polymers of glucosamine based on the β(1–3) or β(1–6) linkages exist, but have gained less synthetic attention. Nevertheless, β(1–6)-linked GlcN oligosaccharides may act as potential antitumor and immunostimulating agents [262] and could become important synthetic targets. The synthesis of a β(1–6)-linked 9mer was demonstrated, using an isopropyl thioglycoside donor [262]. Convergent [262] as well as linear iterative [263] approaches were reported. A block coupling approach enabled access to a collection of free amino or *N*-acetylated structures up to 11mer [264]. Intramolecular glycosylation between a thioglycoside and a free C-6 hydroxy group at the non-reducing end enabled the synthesis of β(1–6)-glucosamine macrocyclic compounds [265]. The iterative glycosylation of *N*-Phth protected 2-deoxy-2-aminothioglycosides allowed for the combinatorial synthesis of short oligoglucosamines having β(1–6) and/or β(1–4)-linkages [263]. Automated solution-phase synthesis of β(1–6)-glucosamine oligosaccharides, ranging from tri- to hexasaccharide, was obtained via iterative electrochemical assembly [266]. A solid-phase automated approach delivered β(1–6)-glucosamine hexasaccharide **87a** and dodecasaccharide **87b** (Scheme 13A) [267]. β(1–3)-Oligomer **90** was prepared as chitin mimetics (Scheme 13B). Despite the subtle differences in the conformational behavior of the two analogues (β(1–3) vs β(1–4) oligomers), it was observed that the β(1–3)-mimetic was still recognized by wheat germ agglutinin and a chitinase enzyme, and could act as a moderate inhibitor of chitin hydrolysis [268].

### Mannose- and rhamnose-based polysaccharides

**Figure 5:**
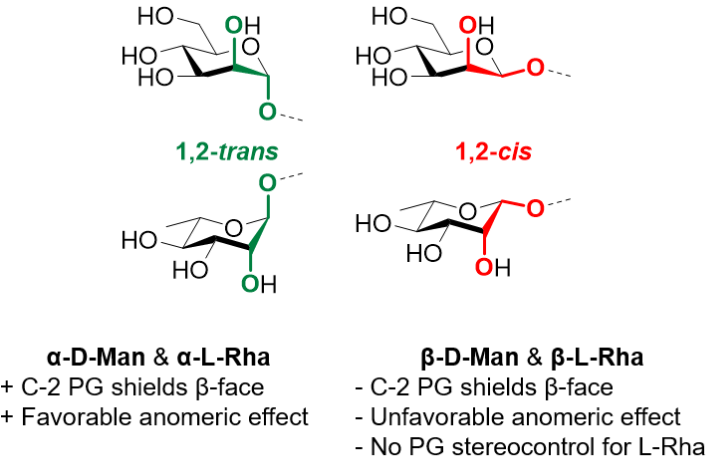
‘Double-faced’ chemistry exemplified for ᴅ-Man and ʟ-Rha. Constructing β-Man linkages is considerably more challenging than constructing α-Man. β-Rha linkages present an additional challenge as PG-directed 1,2-*cis* glycosylations are not viable.

Due to the structural analogies between Rha (both ᴅ and ʟ) and Man, we describe the polysaccharides based on these two units in the same section. From a chemical point of view mannose and rhamnose are ‘double-faced’ monosaccharides, as chemically constructing α-linkage is relatively easy, whereas β-linkages are still considered a major challenge in carbohydrate chemistry (Figure 5). While long α-mannosides could be accessed either by chemical synthesis or by polymerization approaches, only relatively short β-mannan analogues could be synthesized to date. In addition, limited enzymatic approaches exist to assist with the synthesis of mannans and rhamnans.

#### α-Mannans

Mannans are widespread in nature, as constituents of the plant cell-wall (β-mannans, based on the β(1–4)-Man linkage), or on the surface of yeast and pathogens (α-mannans, where α(1–2), α(1–3), and α(1–6) linkages can be found). No enzyme has been reported for the synthesis of α-mannosides. Similarly, polymerization approaches remain to date underrepresented.

α-Mannosides can be reliably prepared through chemical glycosylation, which is in general highly α-stereoselective. Construction of the 1,2*-trans* linkage of α-mannosides is conventionally achieved with [142,157,269], or even without [270], neighboring group participation (e.g., Ac, Bz). The favorable anomeric effect ensures efficient glycosylation reactions. The synthesis of α-mannosides is widely established, allowing the chemical synthesis of the longest polysaccharide to date (i.e., 151mer). To speed up and simplify the solution phase synthesis of long and well-defined polymannosides, iterative coupling strategies are generally adopted [271–272]. Two monosaccharides are coupled yielding a disaccharide, which is parted and converted to the new donor and acceptor. This process is repeated iteratively allowing for an exponential length growth, while maintaining control over the elongation. Beside more common LGs such as phosphates, thioethers, and imidates, ynenoate donors activated using Au(I) catalysis were successfully employed in an iterative strategy, enabling the synthesis of a 32mer [272]. The construction of α(1–6)-mannans (up to 10mer) bearing α(1–2)-Man branches was also accomplished using phosphate, *N*-phenyltrifluoroacetimidate or *n*-pentenylorthoester donors [273–274].

An approach using a (cationic) ROP method using tricyclic orthoester Man BBs considerably reduced the time needed for the stereoselective synthesis of linear α(1–6)-mannans (ranging from 5mer to 20mer) [275]. The biggest advantage of this approach is the easy scalability, up to a gram scale. However, this strategy is limited to one type of linkage (i.e., only α(1–6) linkages) and does not allow for access to mannans with defined length.

**Figure 6:**
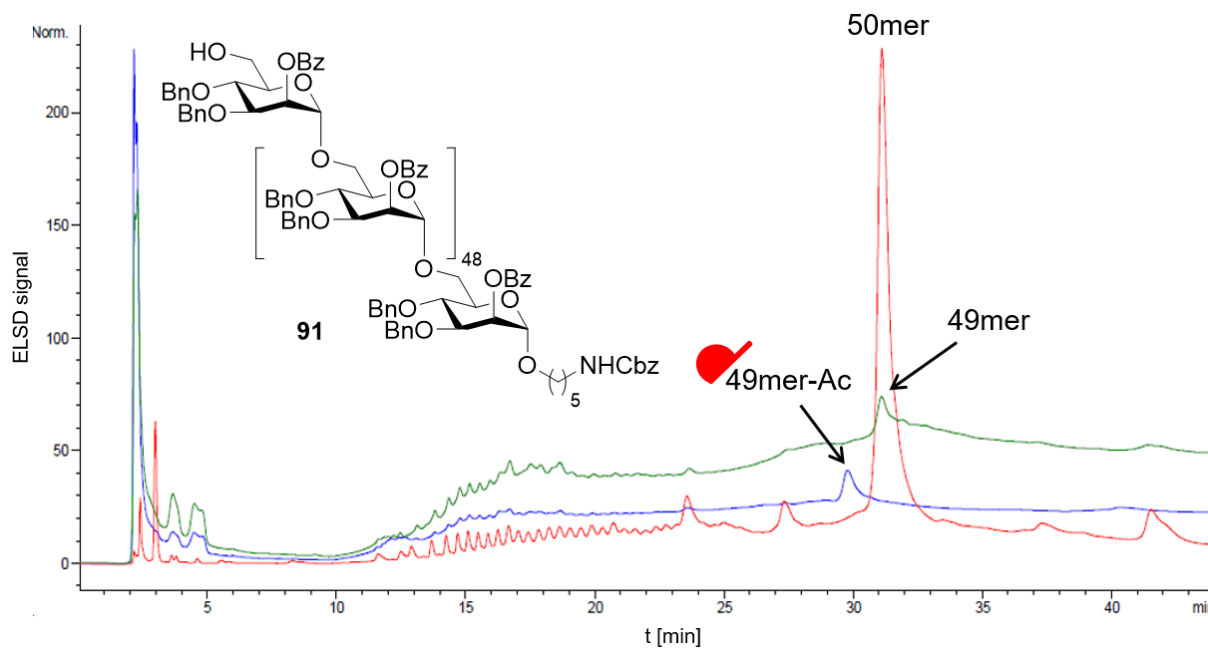
Implementation of a capping step after each glycosylation cycle for the AGA of a 50mer oligomannoside **91**. Uncapped 50mer and 49mer deletion sequence are eluted with virtually the same retention time. The capped 49mer (i.e., C-6 *O*-acetylated) is instead readily separated from the desired 50mer.

**Scheme 14:**
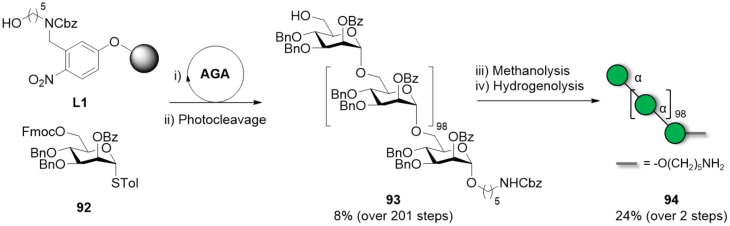
AGA enabled the synthesis of a linear α(1–6)-mannoside 100mer **93** within 188 h and with an average stepwise yield of 98.75%. The AGA cycle includes glycosylation (NIS, TfOH), capping (Ac_2_O), and Fmoc deprotection (piperidine).

Total syntheses of mannose polysaccharides including a 30mer [276], 50mer [277] and 100mer [278] were achieved by AGA. Key optimizations including tuning of the PGs [277], adjustment of coupling time and temperature [278], and introduction of a capping step [279] permitted to drastically improve overall yields and to simplify the purification (Figure 6). The 100mer **93** was obtained with a calculated average stepwise yield of 98.75% (Scheme 14). Long α-mannosides have the advantageous feature of being highly soluble due to the high flexibility of α(1–6)-Man backbone, facilitating the deprotection and purification steps [157].

**Scheme 15:**
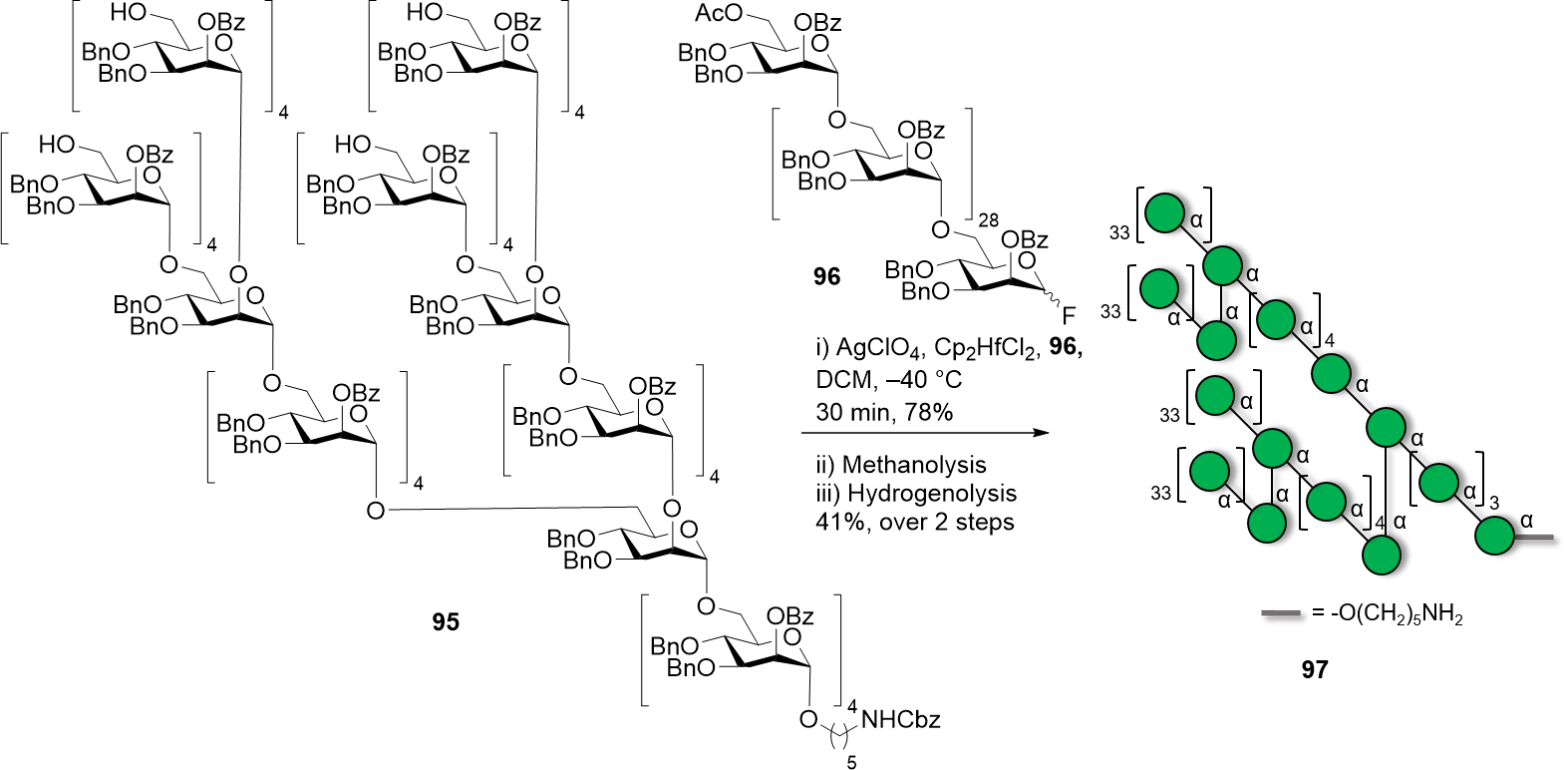
The 151mer branched polymannoside was synthesized by a [30 + 30 + 30 + 30 + 31] fragment coupling. AGA enabled the synthesis of the α-mannoside 30mer donor fragment **96** and the branched 31mer acceptor fragment **95**.

Naturally occurring mannosides, such as lipomannans found on the cell surface of mycobacteria, normally consist of a α(1–6)-mannan core substituted with α(1–2) branches, commonly introduced exploiting orthogonal PGs (e.g., Fmoc and Lev) [269,280–281]. These compounds could be prepared entirely on a solid support, or following a solution-phase fragment (or block) coupling. In this case, the branch produced by AGA, employing a traceless linker [118], was converted into a glycosyl donor and coupled to the mannan acceptor to create a dendron-like structure. To overcome the high steric hindrance of both coupling partners, careful optimization of the LG and the reaction temperature was imperative [118,278,280,282]. A [31 + 30 + 30 + 30 + 30] fragment coupling allowed assembling a 151mer polymannoside **97**, the biggest synthetic polysaccharide obtained to date (Scheme 15). Switching from a *N*-trichloroacetimidate to a less reactive fluoride donor and screening of the suitable activator was key to the success of the coupling. As synthetic polymannosides have now reached the size of macromolecules, new challenges are arising in terms of purification and characterization. Gel permeation chromatography (GPC) and advanced high-resolution MS analysis are being developed to address the need of these new available compounds [278,283].

#### β-Mannans and β-mannuronans

**Figure 7:**
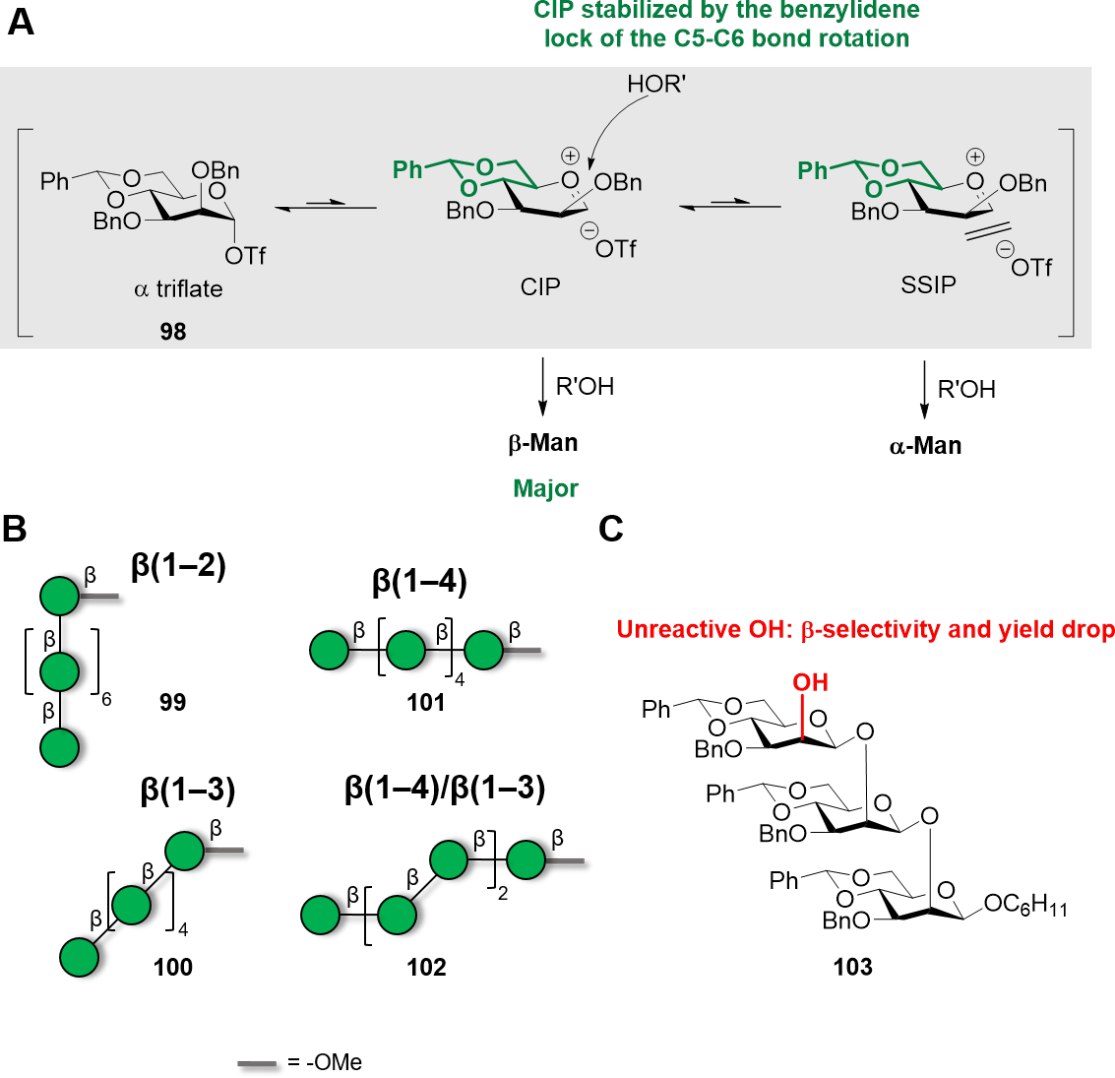
PG stereocontrol strategy to obtain β-mannosides. A) The mechanism of the β-mannosylation reaction is thought to proceed through a contact ion pair (CIP) in which the triflate anion shields the α-face favoring the nucleophile attack from the β-face. The solvent separated ion pair (SSIP) scenario is disfavored due to the benzylidene group locking the C5–C6 bond in the *tg* conformation which destabilizes the oxocarbenium ion. For a thorough explanation of the mechanism we refer to the original publication [289]. B) Examples of β-mannosides synthesized using the 4,6-*O*-benzylidene directed β-mannosylation strategy. C) Chain elongation beyond the trisaccharide level for β(1–2)-analogues presented a significant drop in yield and β-stereoselectivity due to the conformation of acceptor **103**.

In contrast to α-Man linkages, the construction of β-Man glycosidic linkages is a major challenge (Figure 5). Absence of the anomeric effect in the β-product and steric repulsion of the C-2 axial substituent make β-selectivity hard to achieve. Even though, several methods have been developed to construct β-Man glycosidic linkages, the majority of them focused on the construction of a single β-Man linkage [284–285]. Hydrogen-bond-mediated aglycone delivery (HAD) was recently employed to construct a β(1–3) 2-*O*-acetyl-6-deoxy-mannoheptopyranose 4mer with excellent β-selectivity [286]. PG stereocontrol by means of 4,6-*O*-benzylidene is to date the most applied chemical strategy to construct β-mannosides [270]. In a first stage, the donor (either a sulfoxide or a thioglycoside) is pre-activated at low temperature with triflate-based activators. After this step, the acceptor is added, leading to preferential formation of the β-mannoside [287]. The high stereoselectivity is thought to derive from the high stability of the α-triflate intermediate, which dissociates generating a contact ion pair (CIP). In the CIP, the triflate anion shields the α-face, allowing for the preferential attack of the nucleophile from the β-face (Figure 7A). The 4,6-*O*-benzylidene group disfavors the complete dissociation of the α-triflate to the corresponding solvent separated ion pair (SSIP), preventing the formation of the α-Man product [288–289].

Various β-mannosides (up to 8mer) with β(1–2), β(1–4) [290] as well as mixed linkages β(1–3)/β(1–4) [291] were prepared with high stereoselectivity (α:β ratio ca. 1:9) using PG stereocontrol (Figure 7B, **99**–**101**). Interestingly, the synthesis of β(1–2) analogues suffered from a drastic decrease in stereoselectivity (α:β ratio drop to ca. 1:4) and yield as the chain was elongated beyond the 3mer stage (Figure 7C). This observation was ascribed to the characteristic conformation of the protected β(1–2)-trisaccharide acceptor **103**, in which a compact helical structure caused severe hindrance impacting the reactivity and stereoselectivity [290,292]. This issue was not observed with the β(1–4)-mannosides due to their more linear rod-like shape.

Overall, the wide variety of β-mannosides prepared underscored the versatility of the 4,6-*O*-benzylidene directed β-mannosylation to construct the challenging β-Man linkage. Some limitations of the method occur when large Man donors are used, especially when bulky substituents are present at C-3 position (α:β ratio can drop to 1.5:1) [291]. To date, the sulfoxide donor pre-activation protocol and the low temperatures needed (−78 to −60 °C) posed technical challenges to the implementation of this method in AGA. Thus, carboxybenzyl donors bearing a minimally intrusive PG at C-3 were employed in a protocol that does not require pre-activation [293]. However, only trisaccharides with β(1–4)/β(1–3) mixed linkage could be isolated after challenging purification steps, indicating that major limitations still exist for the AGA of β-mannosides.

Since stereoselectivity and yield are sensitive to the acceptor conformation and nucleophilicity, the synthesis of some classes of compounds remain hindered. To circumvent the challenge of β-mannosylation using large donors, an indirect approach was followed to construct β(1–4)-mannosides up to 8mer. The high β-stereoselectivity of β-glucosylation was exploited in a [4 + 4] fragment coupling. The subsequent C-2 epimerization, via an oxidation–reduction step, converted the β-Glc into a β-Man [294]. Similarly, an indirect approach using Glc orthoester donors was applied for the synthesis of a 8mer based on β(1–2)-Man linkages. The newly formed β-Glc was converted to β-Man by Swern oxidation of the hydroxy group at C-2, followed by reduction of the carbonyl group [295]. Other indirect methods to synthesize β(1–2)-mannosides were also reported using donors bearing a ketone functionality at C-2 (ulosyl donors) which require a reduction step to obtain Man [296–297].

**Scheme 16:**
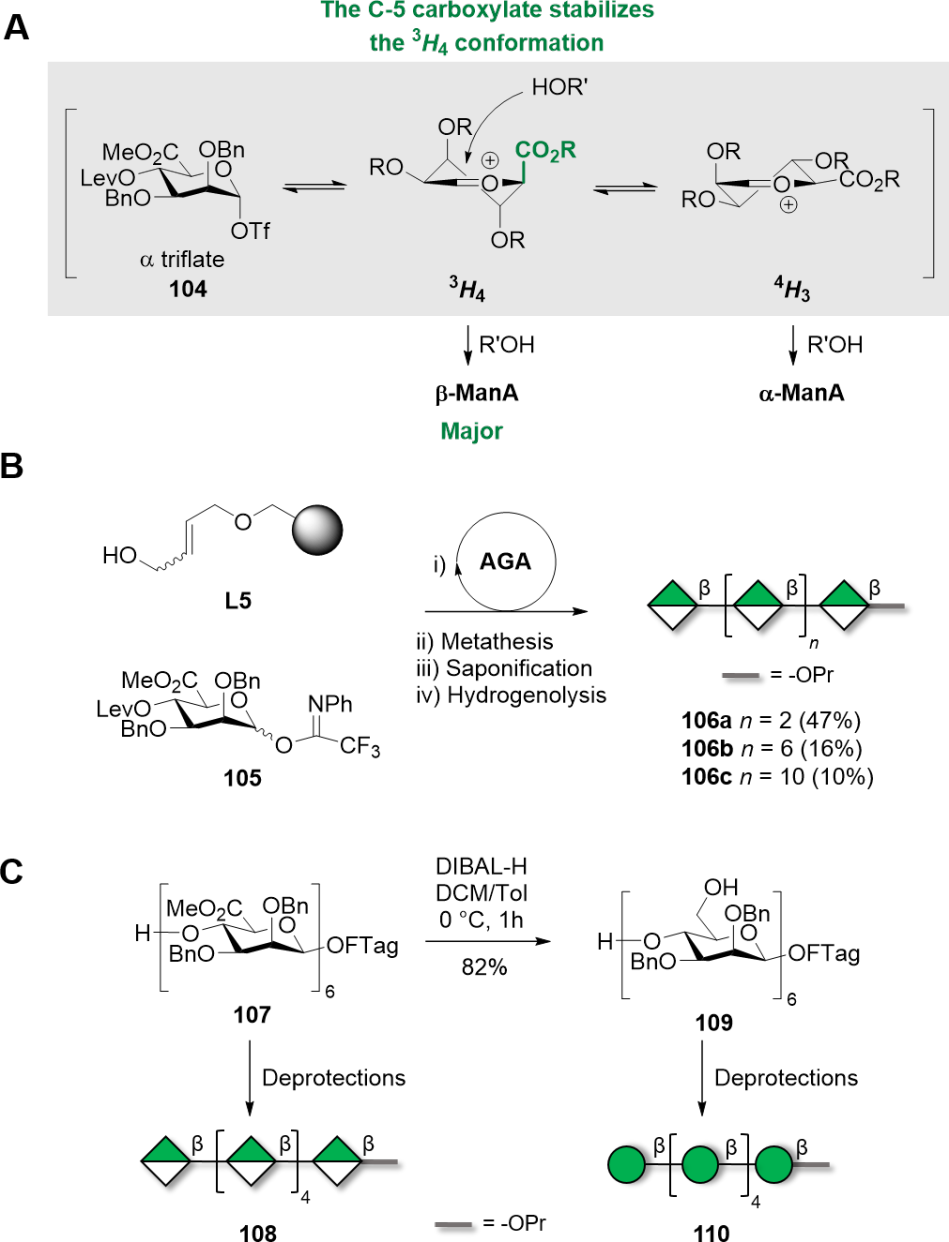
A) Mechanism of 1,2-*cis* stereoselective glycosylation using ManA donors. Once the ManA donor is activated, the C-5 carboxylate stabilizes the oxocarbenium ion in a ^3^*H*_4_ conformation which favors formation of β-ManA linkages. B) Series of β(1–4)-oligomannuronates synthesized using AGA and ManA donors. The AGA cycle includes coupling (TfOH) and Lev deprotection (hydrazine). C) β(1–4)-Man and β(1–4)-ManA oligosaccharides prepared using fluorous-tag (FTag) assisted automated synthesis.

High β-selectivities were observed for mannuronic acid (ManA) donors [298–301]. This result is thought to derive from the unusual ^3^*H*_4_ conformation of the oxocarbenium ion intermediate, stabilized by the *pseudo-*axial C-5 carboxylate (Scheme 16A) [299,301]. The ManA donor enabled the convergent assembly of β(1–4)-ManA oligosaccharides up to 5mer, with a α:β ratio of about 1:10 (in 50–70% yields) [299]. Importantly, yield and β-stereoselectivity were not affected by the size of the coupling partners, a key feature that makes this methodology attractive to access long polysaccharides. This approach was implemented in AGA to generate a collection of mannuronic acid oligomers, ranging from 4mer to 12mer (Scheme 16B, **106a–c**) [302]. Key steps were the choice of the temporary PGs (i.e*.*, Lev), the LG (*N*-phenyltrifluoroacetimidate), activation system (TfOH), and the coupling temperatures (−40 °C) used for BB **105**. An average 90% yield per step with excellent β-stereoselectivity was observed. A solution-phase approach allowed to reduce the amount of required donor (1–2 equiv compared to 9 equiv) and to couple big fragments (i.e., [4 + 4] and [8 + 8] glycosylations) without a significant loss in β-stereoselectivity. A β(1–4)-ManA 16mer was prepared using this highly convergent and iterative approach [303].

The high β-stereoselectivity of ManA donors was capitalized to access β(1–4)-Man 6mer **110**, after reduction of carboxylate moieties of β(1–4)-ManA 6mer **107** (Scheme 16C) [304]. The fast assembly of a β(1–4)-ManA 6mer **107** (75% average yield per step) was achieved by automated solution-phase synthesis with the help of fluorous solid phase extraction (FSPE). Reduction of the carboxylic acid moieties was achieved by diisobutylaluminium hydride (DIBAL-H) treatment.

Enzymatic approaches to access β-mannans are scarce in the literature. The enzymatic synthesis of β(1–4)-mannans (up to DP 7) was reported using glycosynthase mutants of the β-mannanase from *Cellvibrio japonicas.* α-Mannobiosyl fluoride donors were coupled to a Glc acceptor, to obtain the desired product, albeit in a relatively low yield (5% for the 7mer) [305]. Recently, β(1–4)-mannans were synthesized using a thermoactive glycoside phosphorylase from *Thermotoga maritima* that catalyzed the reversible degradation of mannosides by phosphorolysis. An initial screening of temperature and pH, using the α-ᴅ-mannose 1-phosphate donor (αMan1P) and ᴅ-mannose as the acceptor, identified optimal conditions (pH 6.0 and 60 °C), yielding mixtures of oligomers with a DP of 2 to 6. When a β(1–4)-mannoside 6mer was employed as acceptor, higher DP could be reached. The longest oligomers (DP between 7 and 16) precipitated into lamellar crystals with a diffraction pattern typical of mannan I [306].

**Figure 8:**
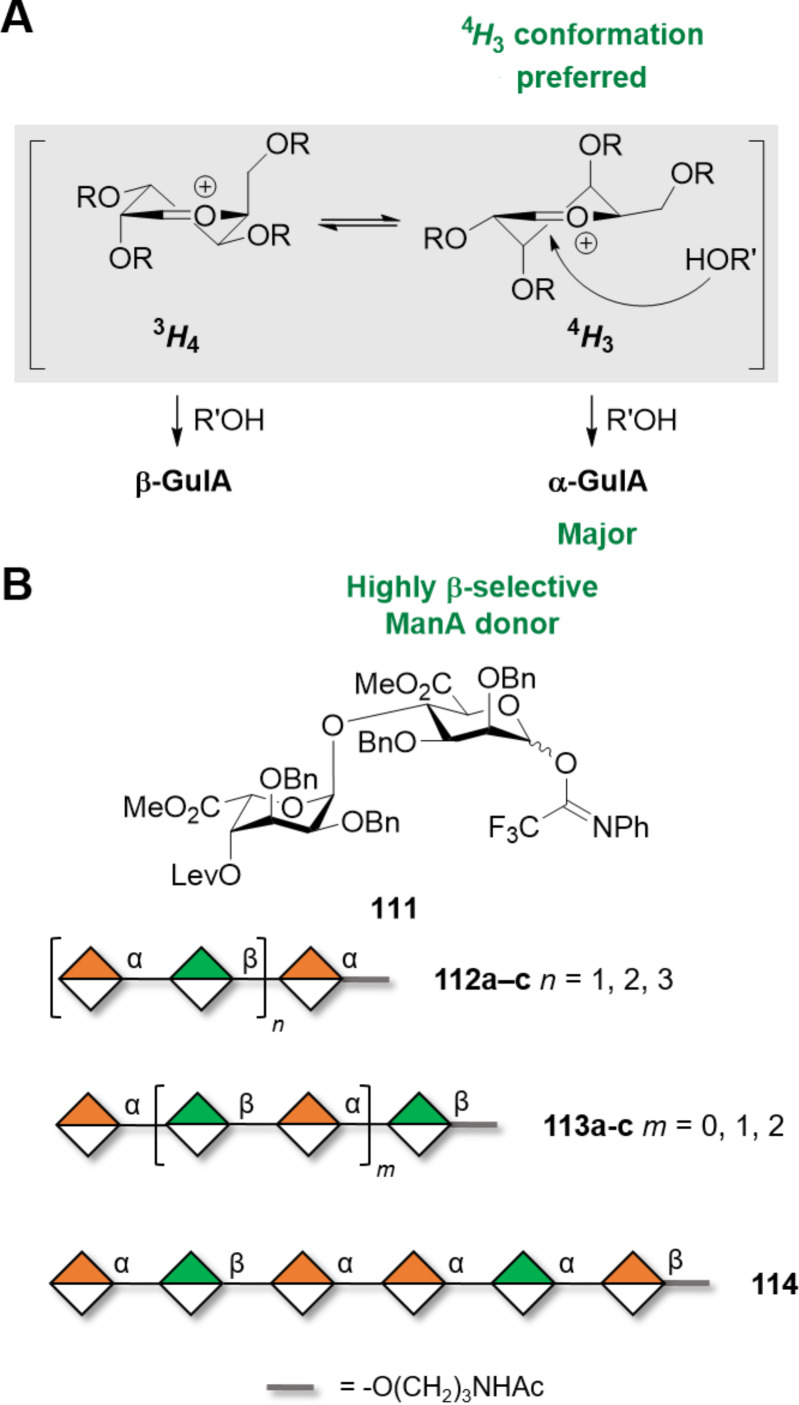
A) The preferred ^4^*H*_3_ conformation of the gulosyl oxocarbenium ion favors the attack of the alcohol from the α-face. B) Key disaccharide BB **111** used for the solution phase synthesis of alginate oligosaccharides and representative structures obtained.

ManA-based polysaccharides are abundant component of alginates, often found as mixed polymers with α(1–4)-ʟ-GulA (i.e., guluronic acid). These naturally occurring anionic polysaccharides are found in algae and some bacteria [307]. The two monosaccharides can be arranged in the polymer in different patterns (i.e., MM, GG, or MG domains where M stands for ManA and G for GulA). To date chemical synthesis is the only way to access well-defined alginate oligosaccharides as enzymatic synthesis has suffered from the complexity of the enzymes involved [308]. The chemical construction of α(1–4)-ʟ-Gul linkages is facilitated by the intrinsic tendency of Gul donors to form 1,2*-cis* glycosidic linkages (α:β ratios >10:1). The ^4^*H*_3_ conformation adopted preferentially by the Gul oxocarbenium intermediate favors the attack of the nucleophile from the α-face, leading to a 1,2*-cis* glycosylation (Figure 8A). In contrast, GulA donors were found to be less α-stereoselective [301,309–310]. Therefore, the most frequently adopted strategies involve highly α-stereoselective glycosylations using Gul donors and subsequent oxidation to obtain GulA [310]. With this strategy, alginate oligomers (i.e., 7mer) with mixed sequence were prepared. The solution phase synthesis was performed by sequential addition of a disaccharide BB **111** with the α(1–4) GulA glycosidic linkage pre-installed (α(1–4) gulosylation followed by C-6 oxidation). The ManA unit provided high β-stereoselectivity (α:β >1:20) (Figure 8B). This strategy, while avoiding late-stage oxidations, suffered from the poor reactivity of the axial OH-4 of GulA resulting in low glycosylation yields for longer analogues (i.e., 30–42%). In addition, the acceptor reactivity was found to be highly sensitive to subtle modifications in remote positions of the acceptor [309,311], highlighting the importance of the – often overlooked – acceptor three-dimensional structure.

#### Rhamnans

Long repetitive polysaccharides constituted of ʟ-Rha (i.e., rhamnans) are found in bacteria, plants, and algae but are absent in animals [312]. The enantiomer ᴅ-Rha is less common, only found in the capsule of some bacterial species. Linear repetitive rhamnans can be classified in type I (alternated α(1–3)/α(1–2) ʟ-Rha residues) or type II (linear α(1–3)-ʟ-Rha residues) and can be variously sulfated.

Very few examples of enzymatic or polymerization methods have been reported to prepare this class of compounds. In contrast, from a chemical standpoint constructing α-rhamnans does not present significant challenges. Similar to Man, Rha donors benefit from the favorable anomeric effect and neighboring group participation, generally achieved with C-2 *O*-acyl groups (e.g., Ac, Bz, Piv), which secures highly stereoselective 1,2*-trans* glycosylations (Figure 5) [270,313–318]. Even in absence of neighboring group participation α-stereoselectivity is achieved [319–320].

**Scheme 17:**
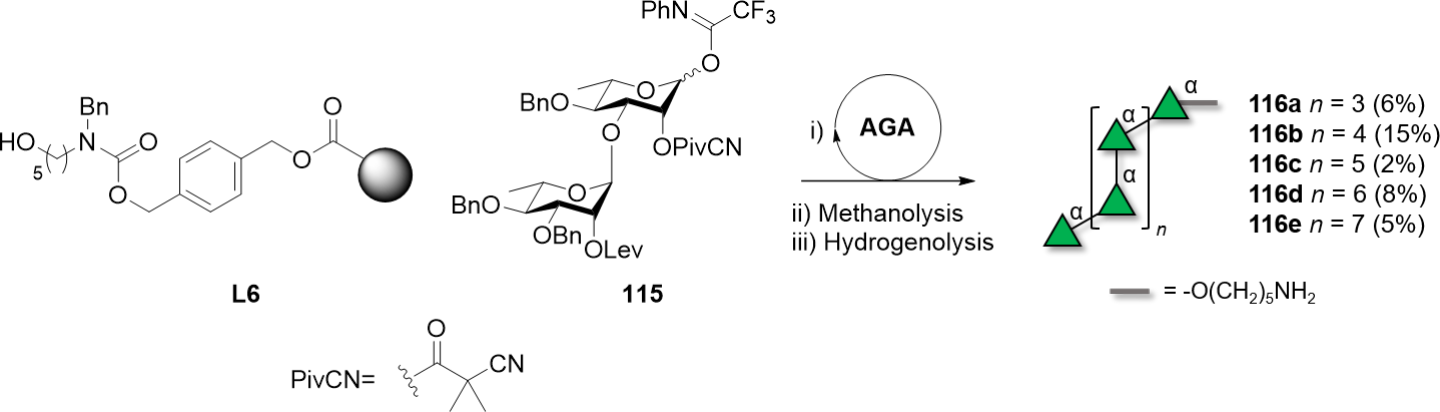
AGA of type I rhamnans up to 16mer using disaccharide BB **115** and CNPiv PG. The AGA cycle includes coupling (TfOH) and Lev deprotection (hydrazine).

α-Rhamnans chemical synthesis suffers from PG migration. This issue has been observed when constructing α(1–3) linkages, since C-2 *O-*Ac groups are prone to migrate from the axial O-2 to the equatorial O-3 of ʟ-Rha [313–314,321–322]. Replacing the Ac group with benzoyl (Bz) [315], pivaloyl (Piv), or cyano-pivaloyl (CNPiv) [313–314] groups prevented acyl migration. In particular, CNPiv was developed to avoid the harsh conditions generally required for Piv removal and could be easily cleaved either by hydrogenolysis [313] or methanolysis [314]. Type I rhamnans up to 16mer (**116a–e**) were prepared by AGA using disaccharide BB **115** (Scheme 17) [314]. Type I and II α-rhamnans were also successfully assembled following solution-phase approaches by stepwise elongation of the rhamnan chain using orthogonally protected disaccharide BBs. The range of orthogonal PGs used (i.e., PMB, Bz, Nap, Bn) suggested the possibility to generate rhamnan structures with different sulfation patterns [315–316]. Orthogonality of PGs was also exploited to introduce β(1–3)-GlcNAc branches on a type I rhamnan 8mer backbone, common in bacterial α-rhamnans [317].

In analogy to mannose, β-rhamnosylation is a highly challenging reaction. However, while the β-mannosylation reaction can benefit from 4,6-*O*-benzylidene PG stereocontrol, β-rhamnosylation cannot [323]. To date, only few reports of successful β-rhamnans synthesis are available, mostly limited to short oligosaccharides [324].

#### Polysaccharides based on a mixed mannose–rhamnose backbone

The lack of enzymatic approaches to construct the often unique linkages found in bacterial polysaccharides makes chemical synthesis the only amenable way to access these molecules. Generally, the repeating unit containing the challenging linkage is prepared and used in iterative glycosylation approaches enabling exponential growth, while maintaining control over the polysaccharide length.

**Figure 9:**
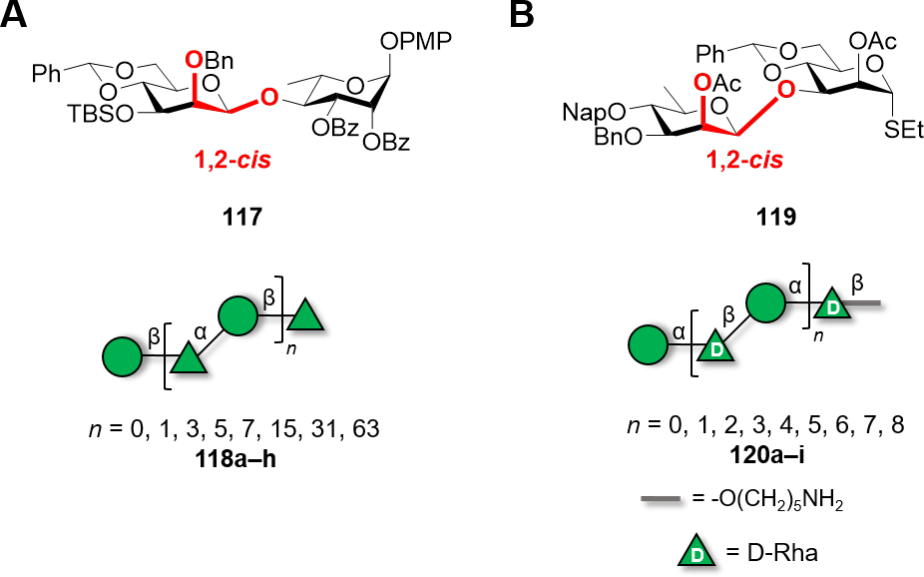
Key BBs for the synthesis of the *O*-antigen of *Bacteroides vulgatus* up to a 128mer (A) and the CPS of *Clostridium bolteae* up to 18mer (B). The most challenging 1,2-*cis* glycosidic linkage is installed in the key BBs and highly stereoselective 1,2-*trans* α-rhamnosylation or α-mannosylation were exploited for elongation.

Recently, the synthesis of large bacterial polysaccharides containing ᴅ-Man, and ᴅ/ʟ-Rha mixed structures was reported [325–326]. The *O*-antigen of *Bacteroides vulgatus* consisted of alternating α(1–3)-ʟ-Rha and β(1–4)-ᴅ-Man linkages. The efficient α-rhamnosylation reaction was exploited to elongate the chain by iterative glycosylations, while the challenging β(1–4)-Man was installed at the disaccharide level (**117**) using the 4,6-*O*-benzylidene directed β-mannosylation (Figure 9A). The exponential growth, up to the 128mer (**118a–h**), was enabled by *o*-alkynylbenzoate donors [327] and the use of two orthogonal PG at the reducing and non-reducing end (i.e., *p*-methoxyphenyl (PMP) and *tert*-butyldimethylsilyl (TBS) groups, respectively). The glycosylation reactions proved robust, with only a slight drop in yield (i.e., 74% compared to ca. 90%) for the [64 + 64] glycosylation. Issues during the final deprotection steps, due to poor solubility and reducing end degradation [294], lowered the isolated yield to 15% for the longest analogues [325].

The capsular polysaccharide (CPS) of *Clostridium bolteae,* consisting of alternating α(1–4)-ᴅ-Man and β(1–3)-ᴅ-Rha, was targeted to showcase a methodology to construct β-ᴅ-Rha linkages. Similar to the above approach, the disaccharide BB **119** including the challenging β(1–3)-ᴅ-Rha glycosidic linkage was initially prepared (Figure 9B). The efficient α-mannosylation reaction was exploited to further elongate the chain in a sequential manner. Three different strategies were screened for the construction of the dimer: i) β-rhamnosylation through a pre-activation strategy, ii) 4,6-*O*-benzylidene directed β-mannosylation and subsequent C-6 deoxygenation to provide ᴅ-Rha, iii) indirect approach using 6-deoxy-ᴅ-Glc in a 1,2-*trans* glycosylation and subsequent C-2 epimerization to give ᴅ-Rha. Strategies ii) and iii) successfully formed the β(1–3)-ᴅ-Rha linkage of the disaccharide BB. A series of sequential glycosylations enabled the solution-phase assembly of oligosaccharides up to 18mer (**120a–i**) in excellent yields (80–90% per coupling). The deprotection (i.e., methanolysis and hydrogenolysis) proved to be challenging for longer analogues due to the degradation of the oligosaccharides in the acidic solvent chosen for hydrogenolysis. A neutral solvent mixture permitted to obtain the compounds, even though in low yield compared to shorter analogues [326].

### Galactose-based polysaccharides

Enzymatic and polymerization approaches are not so common for galactopyranose-based polysaccharides and are limited to the construction of single glycosidic linkages [328]. In contrast, bacterial enzymes capable of constructing galactofuranose-based polysaccharides have been widely studied and offer the unique possibility to tune the chain length. Additionally, chemical synthesis can reliably afford both α-Gal and β-Gal polysaccharides.

#### β-Galactans

Galactans and arabinogalactans, together with arabinans, are the three types of side chains in the pectin RG-I domain [99,329–330]. The backbone of type I galactans is made of β(1–4)-linked Gal residues, while type II galactans are highly branched polysaccharides composed of β(1–3)- or β(1–6)-linked Gal residues [99].

**Figure 10:**
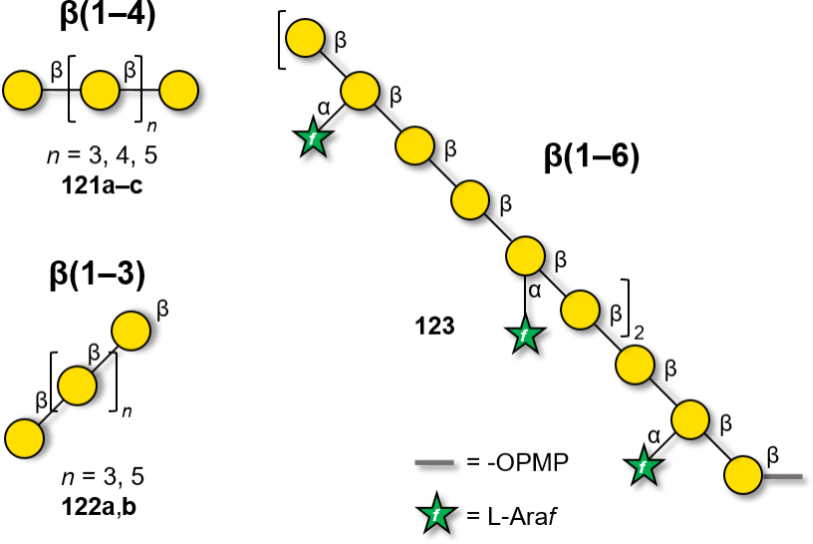
Examples of type I and type II galactans synthesized to date.

β-Galactosylation is easily achieved by neighboring group participation. The main challenge of type I galactans is the low reactivity of the axial hydroxy group at C-4 of the Gal residues. Type I galactans up to 7mer (**121a–c**) have been prepared employing C-2 *O*-Piv or *O*-Ac PG and either *n*-pentenyl or thioglycoside LG (Figure 10) [331–333]. Steric bulk around the unreactive C-4 OH was minimized by using Bn or allyl (All) ethers at C-3 and C-6 positions [331]. Removal of Piv, despite requiring harsh conditions (LiOH in MeOH under reflux or Et_4_NOH in THF under reflux), yielded the desired product in good yield (70–90%). In contrast, removal of allyl (All) PG was more challenging, requiring extensive optimization [331]. The use of Nap to mask the hydroxy groups at C-4 or C-6 allowed for the late-stage installation of arabinan side chains [332]. AGA was employed to assemble a collection of type I galactans using three different Gal BBs equipped with Fmoc as a temporary PG to allow for chain elongation. Lev could be selectively removed to install ʟ-arabinose branches at the C-3. The low reactivity of the hydroxy group at C-4 required a double cycle of glycosylation and negatively impacted the final yields [334].

The easy formation of β-Gal linkages and the excellent reactivity of the primary hydroxy group at C-6 considerably simplified the synthesis of type II galactans. Solution-phase synthesis provided long β(1–6)-galactans, bearing α(1–2)- or α(1–3)-linked ʟ-arabinofuranose side-chains [335–338]. A stepwise elongation (i.e., [4 + 12] then [4 + 16]) proved successful to construct a 20mer β(1–6)-galactan, bearing arabinose side chains [338] (**123**, Figure 10).

Only shorter synthetic oligomers based on a β(1–3)-Gal backbone could be obtained (**122a,b**). A collection of linear and branched type II galactans was synthesized by solution-phase methods. To minimize PG migration, the use of thioglycoside disaccharide donors bearing a participating Piv ester at C-2 position was the key [339]. Similar structures were obtained by AGA [340]. In both cases, orthogonal PG were necessary for the installation of either β(1–6)-Gal or α(1–3)-Ara*f* branches.

#### α-Galactans and α-galactosaminogalactans

**Figure 11:**
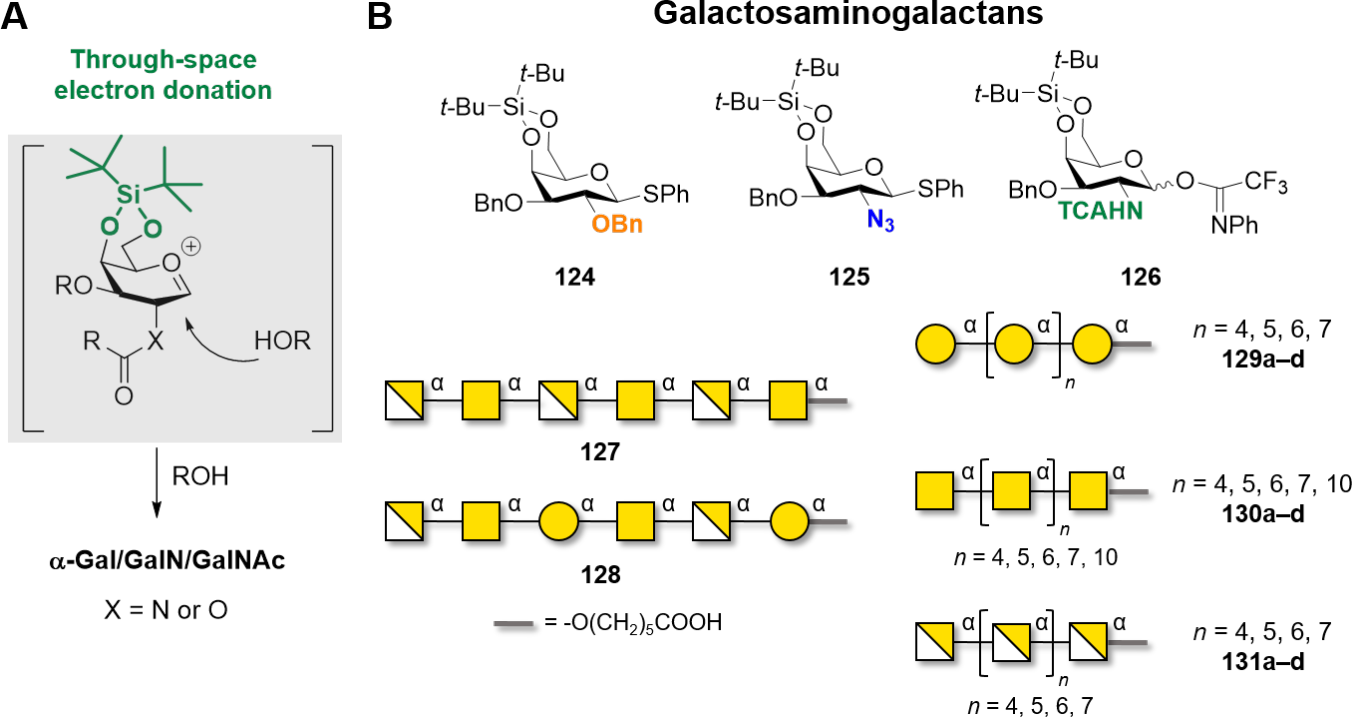
A) The DTBS PG stabilizes the ^3^*H*_4_ conformation of the Gal oxocarbenium ion favoring the attack of the alcohol from the α-face in a S_N_1 manner. B) Key BBs used in the synthesis of galactosaminogalactans bearing the α-directing DTBS PG.

In contrast to the challenging formation of other 1,2-*cis* linkages, α-galactosylation can be reliably obtained using Kiso’s di-*tert*-butylsilylene (DTBS)-directed α-stereoselective methodology (Figure 11A). Upon activation, the galacto-type oxocarbenium adopts a ^4^*H*_3_ conformation, preferentially attacked from the α-face in a S_N_1 mechanism due to the steric bulk of the DTBS group. The ^4^*H*_3_ conformation is stabilized by through-space electron donation from the DTBS group. Complete α-stereoselectivity is often preserved, even in presence of C-2 participating PGs [341–342].

This methodology was elegantly implemented for the synthesis of galactosaminogalactans, linear heteropolysaccharide found in the cell-wall of the fungus *Aspergillus fumigatus* (Figure 11B) [343–344]. Natural galactosaminogalactans are composed of Gal, GalN, and GalNAc arranged in a random pattern impeding their isolation from natural sources. A sequential strategy using BBs **124**, **125**, and **126** afforded a collection of α(1–4)-Gal, α(1–4)-GalN, α(1–4)-GalNAc, including mixed structures. The use of the highly reactive *N*-phenyltrifluoroacetimidate glycosyl donors **126** proved to be crucial to generate long oligosaccharides. Chain elongation was achieved by DTBS removal and selective benzoylation of the OH at C-6, yielding the free C-4 hydroxy group to be used as acceptor in the next glycosylation. Good yields (80–90%) and excellent α-stereoselectivities were obtained for the construction of oligomers up to 12mer (**127**, **128**, **129a–d**, **130a–d**, **131a–d**). The deprotection of α(1–4)-Gal 8mer and 9mer suffered from low yields (20–30%), due to low solubility in water. Higher yields were obtained for more soluble analogues [343–344].

**Figure 12:**
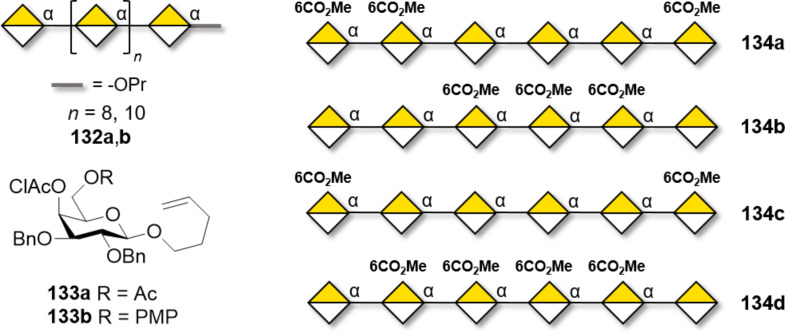
Homogalacturonan oligosaccharides synthesized to date. Access to different patterns of methyl-esterification could be controlled using BBs **133a** and **133b** bearing different C-6 PGs (i.e., Ac or PMP).

The pectin domain homogalacturonan (HG) consists of a linear backbone of α(1–4)-GalA residues in which the C-6 carboxylate moiety can exist as methyl ester, generating different patterns [99,329–330]. While the stereoselective construction of the α(1–4) linkages did not pose a significant challenge, the poor acceptor reactivity due to the presence of the carboxylate moiety often translated into low yield [99]. HG analogues (up to a 12mer, **132a**,**b**) were successfully synthesized by solution-phase synthesis (Figure 12). Solvent participation with Gal fluoride donors (activated with SnCl_2_, AgClO_4_) ensured high α-stereoselectivities. The Gal residues were converted to GalA by selective hydrolysis of the Ac ester at C-6 and a two-step oxidation (i.e., Swern oxidation followed by treatment with NaClO_2_ in aqueous acidic conditions) [345–346]. An alternative oxidation protocol (Dess–Martin periodinane/NaClO_2_) was applied to synthesize HG fragments with different methylation patterns (**134a–d**). Two orthogonal PGs (Ac and PMP) were installed at C-6 of the α(1–4)-Gal fragments, allowing for selective oxidation (Figure 12). Methylation (with TMSCHN_2_) or benzylation (with PhCHN_2_) of the carboxylic acid allowed to differentiate between the carboxylic acids and obtain the methyl ester or the carboxylic acid, respectively [347–348]. Overall, while requiring more PG manipulations, the post-glycosylation oxidation strategy proved to be higher yielding compared to a procedure using GalA donors [349].

#### Galactofuranose-based polysaccharides

The cell wall of mycobacteria contains galactofuranose (Gal*f*)-based polysaccharides that play various functional roles based on their length [350]. These polymers are biosynthesized in a length-controlled manner by complex enzymatic machineries. However, to date, the molecular mechanism for chain length control remains poorly understood [351].

**Figure 13:**
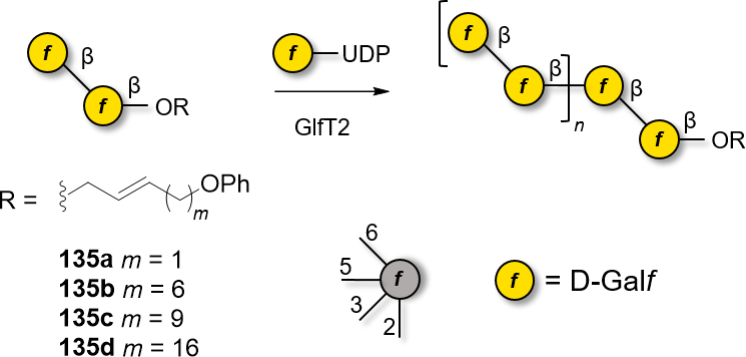
GlfT2 from *Mycobacterium tuberculosis* catalyzes the sequential addition of UPD-Gal*f* donor to a growing galactan chain composed of alternated β(1–5)- and β(1–6)-linked ᴅ-Gal*f*.

The galactan found in *Mycobacterium tuberculosis* is composed of 20–40 Gal*f* residues connected via alternated β(1–5)- and β(1–6)-linkages. The in vitro enzymatic synthesis of these galactans has been reported using galatofuranosyltransferase (GlfT2) from *Mycobacterium tuberculosis,* UPD-Gal*f* donor, and acceptor **135c** (Figure 13). The resulting polysaccharide showed a product distribution unbalanced towards larger polymers (up to 27mer), with a narrow distribution centered on the 21mer. The nature and length of the lipidic part was a key parameter to control the in vitro polymerization. While **135a** and **135b** did not function as efficient acceptors, longer galactans with distribution centered on the 41mer could be obtained tuning the lipid length (acceptor **135d**) [352]. The mechanism of galactan length control may differ consistently between the in vitro and in vivo setting as the interplay between the GlfT2 enzyme, the plasma membrane and other proteins involved in transport across the membrane play a crucial role in length control [353–354]. Recently, it was reported that a mutation on the GlfT2 enzyme enabled the regulation of the galactan chain length (4mer or 11mer with a relatively narrow distribution) [350]. These examples suggest a potential avenue to produce length-controlled polysaccharides via glycoengineering [351,354–355].

**Figure 14:**
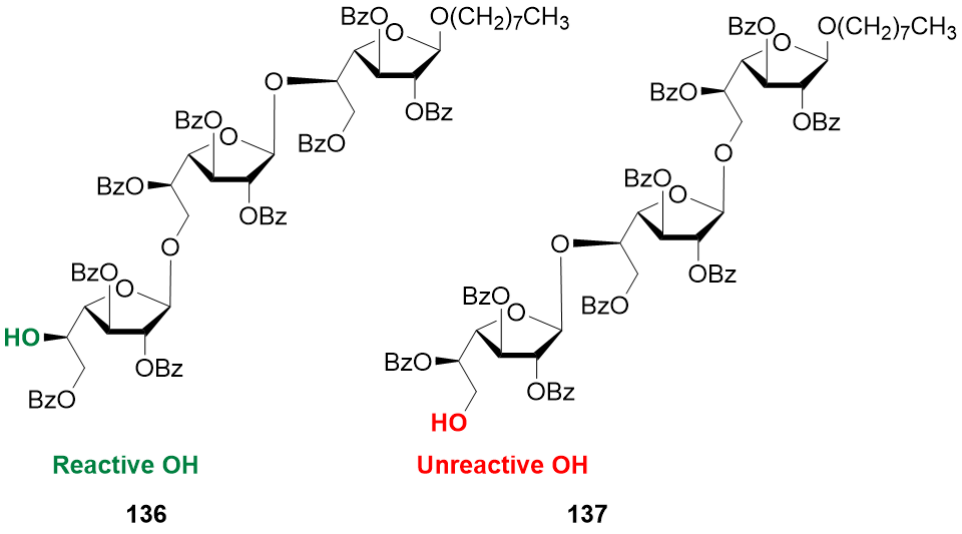
The poor reactivity of acceptor **137** hindered a stepwise synthesis of the linear galactan backbone at the 3mer stage. In contrast, acceptor **136** proved to be more reactive.

The main challenges associated to the chemical construction of alternated β(1–5)- and β(1–6)-linked ᴅ-Gal*f* residues are the complexity of Gal*f* BB synthesis. Especially for Gal*f*, the lower thermodynamic stability compared to its pyranosic counterpart, makes BBs synthesis more complex [356–359]. Common approaches to access Gal in the furanose form involved dithioacetal cyclization using iodine and an alcohol [358] or a recently reported pyranoside-into-furanoside (PIF) rearrangement [360]. Frequent O-5→O-6 ester migration is observed in basic media. The migration could be reduced by using morpholine, instead of piperidine, during the Fmoc deprotection permitting the synthesis of a 7mer upon stepwise chain elongation [358–359]. Other approaches employing Lev as a temporary PG yielded a 14mer galactan [361]. Interestingly, while a good reactivity of acceptor **136** was observed, the poor reactivity of acceptor **137** hindered the stepwise synthesis of the linear backbone, already at the 3mer stage (Figure 14). This poor reactivity was ascribed to the unfavorable conformation adopted by **137**. This problem was solved by building the galactan backbone from the non-reducing end to the reducing end [358].

**Scheme 18:**
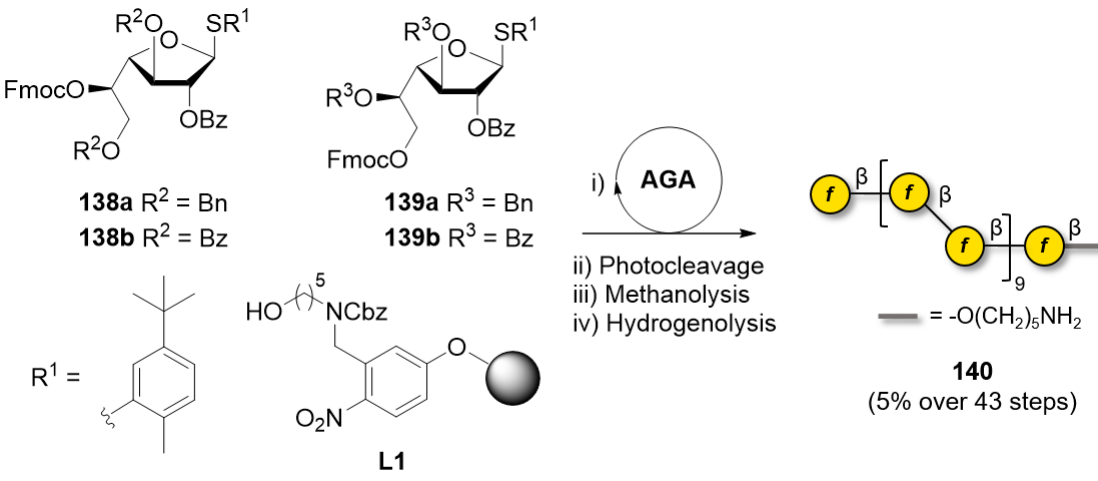
AGA of a linear β(1–5) and β(1–6)-linked galactan 20mer. The AGA cycle includes coupling (NIS/TfOH), capping (Ac_2_O) and Fmoc deprotection (DBU).

Galactan fragments, up to 20mer, were prepared by AGA (Scheme 18). When BBs **138a** and **139a** were used, several problems were encountered such as poor solubility, aggregation of the growing chain on the solid support and instability. Switching to BBs **138b** and **139b** bearing disarming Bz groups solved the issue and enabled the assembly of galactans up to 20mer **140** [362].

### Fucose-based polysaccharides

Fucans are a class of ionic polysaccharides present on the cell wall of marine brown algae, mainly constituted of repeating ʟ-fucose units [363]. They are heavily sulfated and connected through α(1–3) or α(1–4) linkages [364]. Several orthogonal PGs are required to access different sulfation patterns present in fucans. Long structures (up to 16mer) with α(1–3) connectivity were obtained by iterative couplings of dimers and tetrasaccharides units [365]. Donors bearing 4-*O*-benzoyl, in combination with the 3-*O*-Ac and 2-*O*-Bn groups, enhanced the α-selectivity through remote participation. To date, only short tetramers containing both types of α-linkages have been reported [366].

### Arabinose-based polysaccharides

Arabinans, repetitive polysaccharides containing arabinofuranose (Ara*f*), are major constituents of both plant cell-wall and of mycobacterial cell-wall, with the important difference that plants contain ʟ-Ara*f,* while bacteria ᴅ-Ara*f* [367]. Mycobacterial arabinans are composed of a linear backbone of α(1–5)-ᴅ-Ara*f* with α(1–3) branches, which can be further elongated with linear α(1–5)-ᴅ-Ara*f* residues. Similarly, plant arabinans consist of α(1–5)-ʟ-Ara*f* bearing α(1–3) and/or α(1–2) branches.

A variety of strategies have been reported to synthesize these compounds. Most of them show several common features. Bz groups are the most commonly used neighboring participating groups to ensure stereoselectivity, affording the linear α(1–5)-ᴅ-Ara*f* backbone. Convergent strategies are commonly employed [368]. As an alternative approach, cationic ROP using 1,2,5-orthobenzoate BBs and Lewis acid catalysis (i.e., SnCl_4_ or TMSOTf) could generate mixtures of linear α(1–5)-arabinans (up to 25mer) [369–371]. Using 1,2,5-orthopivaloate BBs, linear α(1–5) arabinans with DP up to 91 could be prepared [372]. Tuning the polymerization conditions gave mono-, di-, and trisaccharide thioglycoside Ara*f* BBs, that were used to assemble defined linear arabinans (up to 8mer) [373].

When constructing branched arabinans, convergent approaches involving fragment coupling, rather than sequential elongation, are preferred. Steric hindrance at the arabinan acceptor was found to be the limiting factor during the installation of the α(1–3)-arabinan branches. Small Ara*f* donors enabled the installation of C-3 branches in a 12mer [374–375]. Larger structures (up to 22mer) were synthesized by convergent fragment coupling [376–377]. Key to the success of these syntheses was the early stage installation of the α(1–3) branches and the single β(1–2)-linked Ara*f* at the non-reducing end. Trichloroacetimidates were chosen as reactive LGs for the most challenging couplings (i.e., [12 + 3 + 3], and [12 + 5 + 5]). To avoid the removal of a large number of Bn groups at the last stage of the synthesis, Bz groups were chosen as the sole PGs for the fragments [376]. The reactivity of Ara*f* permitted the implementation of milder glycosylation conditions; 1,2-propargyl orthoester donors and alkynyl carbonate donors, activated using a Au(I) catalyst in presence of AgOTf, were employed in the convergent synthesis of a 25mer. The glycosylation could be carried out at room temperature [368,378–380]. Shorter branched structures (up to 6mer) could be assembled by AGA using thioglycoside donors [281,381].

Fewer examples exist for the chemical synthesis of ʟ-arabinans. A convergent assembly of a α(1–5)-linked 8mer was accomplished with ʟ-Ara*f N*-trichloroacetimidate donors. Regioselective glycosylation of the primary alcohol at C-5 on partially protected acceptors was possible, simplifying BB preparation. However, this reaction was shown to be highly acceptor-dependent and worked best only for Ara*f* acceptors [382–383].

**Figure 15:**
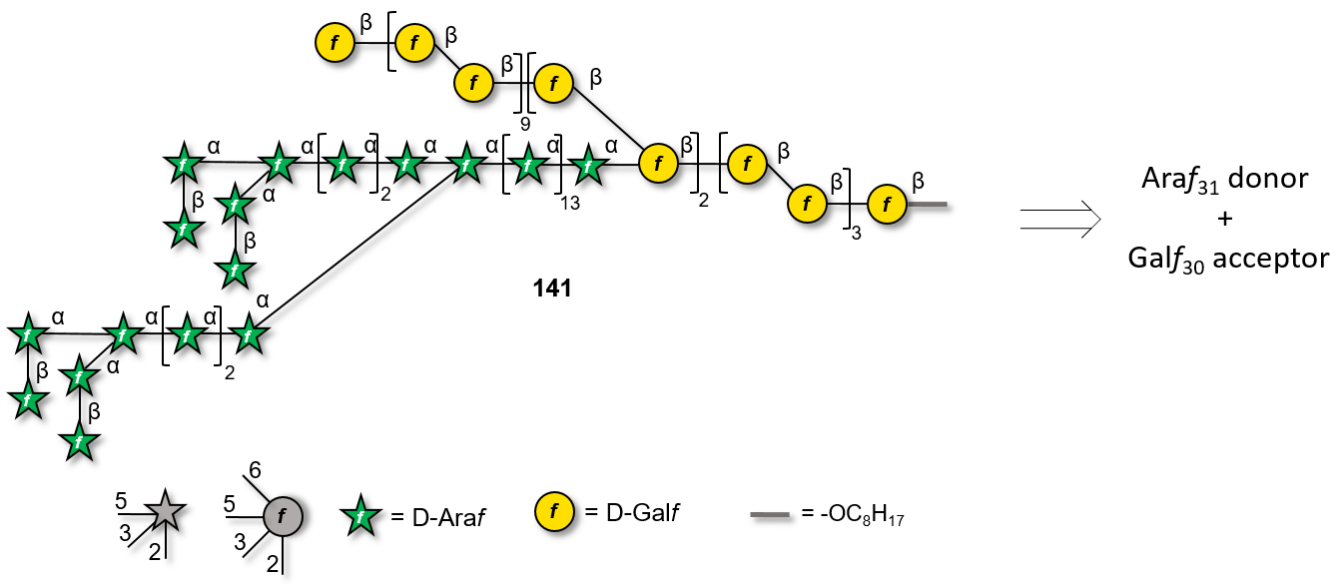
The 92mer arabinogalactan was synthesized using a [31 + 31 + 30] fragment coupling between a 31mer arabinan donor (Ara*f*_31_) and a 30mer galactan acceptor (Gal*f*_30_).

Recently, the synthesis of the whole complex arabinogalactan polysaccharide 92mer was reported. A linear 30mer backbone of alternated β(1–5)- and β(1–6)-linked ᴅ-Gal*f* residues bearing two branched 31mer arabinan was assembled in a convergent approach. Two equivalents of a branched arabinan donor (bearing α(1–5) and α(1–3) linkages) were coupled to a linear galactofuranose (30mer) backbone acceptor in a [31 + 31 + 30] glycosylation step (Figure 15).

**Scheme 19:**
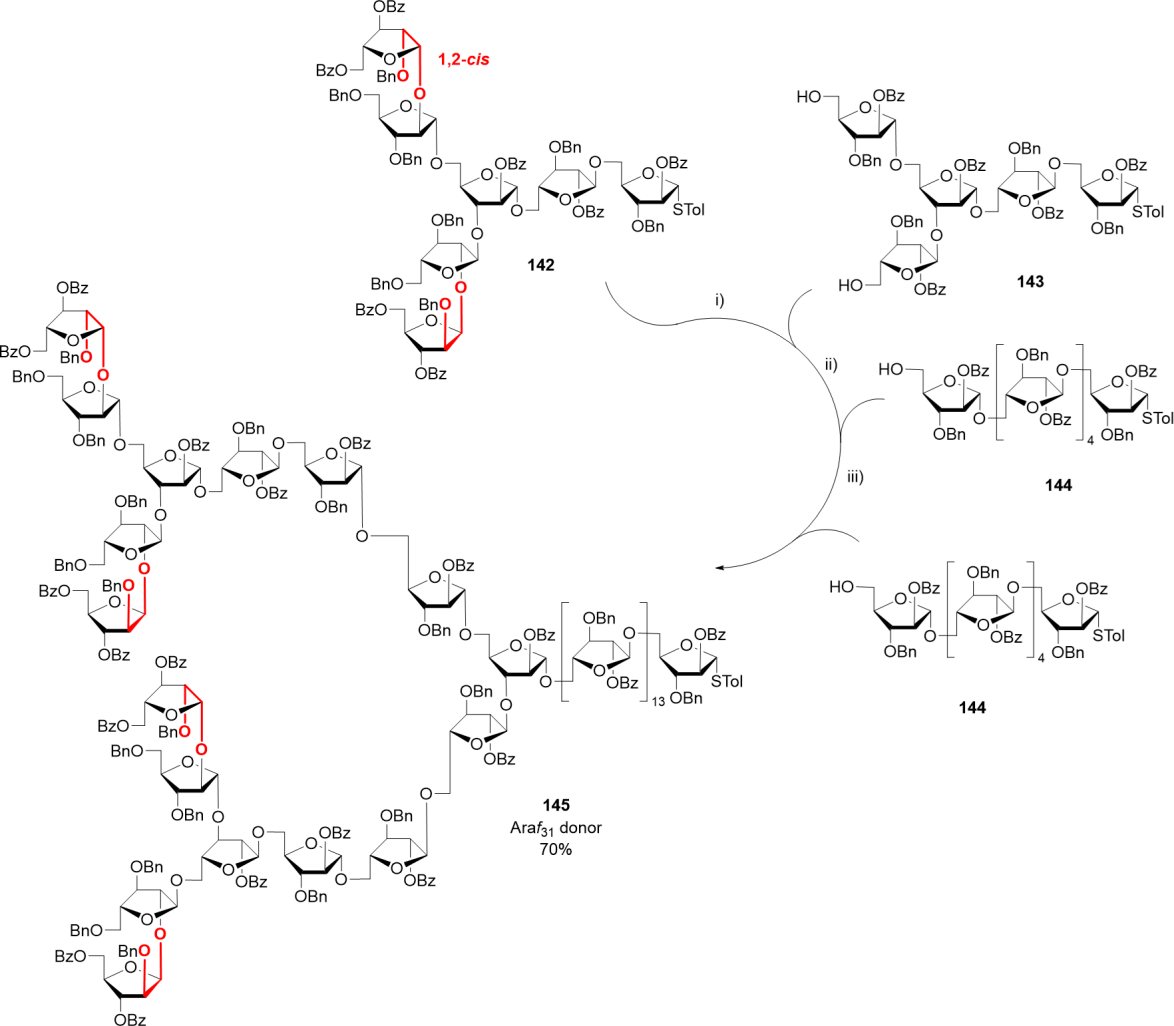
Synthesis of the branched arabinofuranose fragment using a six component one-pot synthesis. i) TTBP, 4 Å MS, DCM, *p*TolSCl, AgOTf then **143**, −78 °C to room temperature. ii) *p*TolSCl, AgOTf then **144**, −78 °C to room temperature. iii) *p*TolSCl, AgOTf then **144**, −78 °C to room temperature.

The three fragments were assembled following one-pot protocols, in which the donor was pre-activated at low temperature (stoichiometric *p*TsCl/AgOTf at −78 °C) and then the acceptor was added [154]. Upon completion of the reaction, the newly formed compound was subjected to the same pre-activation protocol and employed in the following step without intermediate purification. In this way, the alternated linear β(1–5)- and β(1–6)-linked ᴅ-Gal*f* 30mer fragment was quickly obtained via a five-component one-pot glycosylation [6 + 6 + 6 + 6 + 6]. Each 6mer was itself generated via a six-component one-pot protocol. Similarly, branched arabinofuranose fragment **145** was assembled following a six-component one-pot glycosylation [7 x 2 + 5 + 6 + 6] (Scheme 19). To avoid late-stage installation of challenging linkages, the β(1–2)-Ara*f* terminal residues were installed at an early stage using a 3,5-TIPDS protected (3,5-O-tetraisopropyldisiloxanylidene) Ara*f* donor [384].

The three fragments were finally connected using the benzenesulfinylmorpholine/triflic anhydride (BSM/Tf_2_O) activator system. Despite the impressive size of the molecule, no issues were encountered in the final deprotection steps (75% yield), permitting to obtain 92mer arabinogalactan polysaccharide **140** [385].

### Glycosaminoglycans (GAGs)

**Figure 16:**
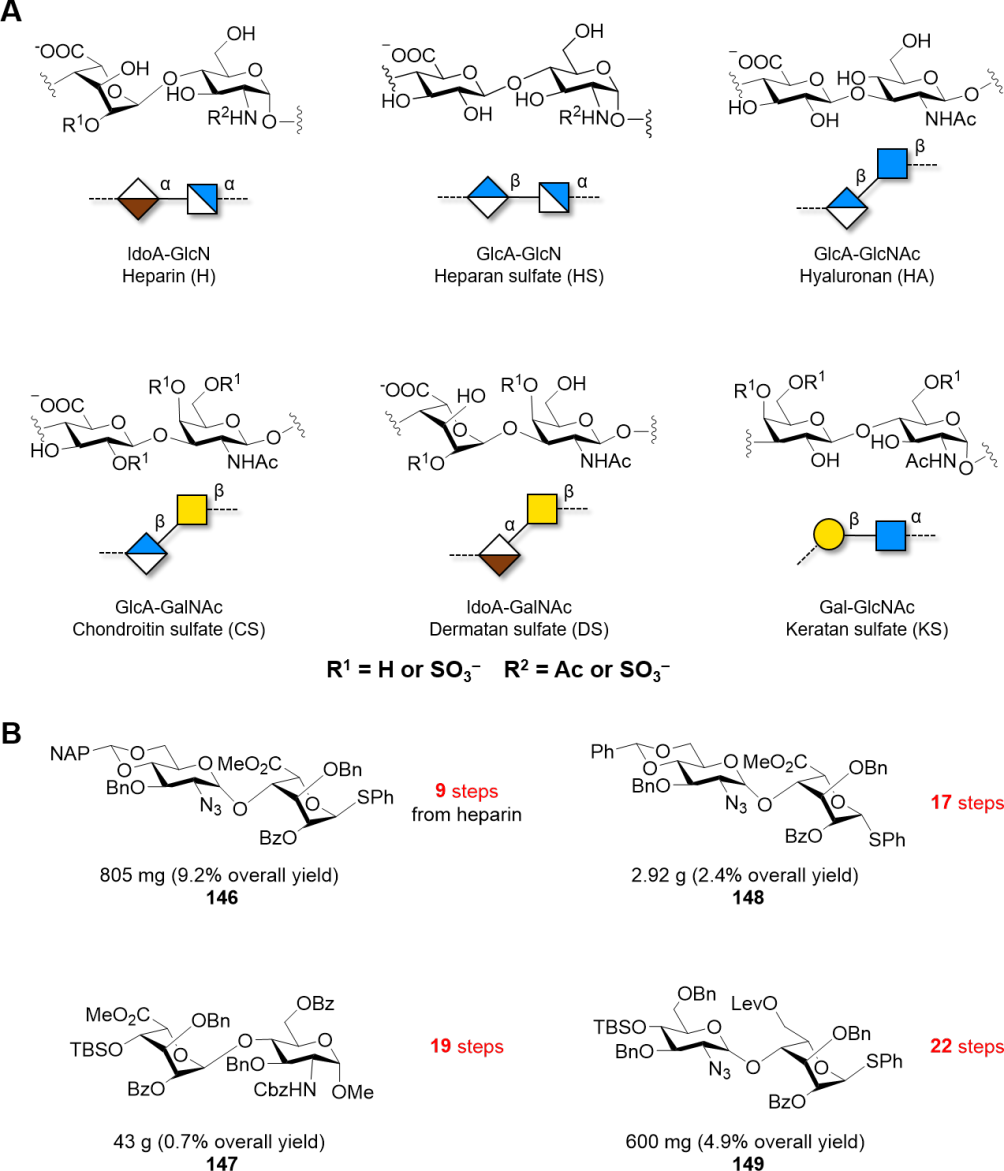
A) Chemical structure and SNFG of the representative disaccharide units forming the GAG backbones, and B) representative examples of synthetic disaccharides used for the preparation of HS analogues.

Ionic polysaccharides are ubiquitous in living organisms where they regulate a multitude of cell functions. The ionic moieties control the shape of such compounds by promoting repulsive and attractive interactions [9]. Glycosaminoglycans (GAGs) are the major structural components of the extracellular matrix (ECM) in mammals. GAGs are linear, negatively charged polysaccharides composed mainly of repeating disaccharide units (Figure 16A) [386]. The anionic backbone, bearing sulfate moieties (except for hyaluronans), is responsible for their interactions with a plethora of proteins and the regulation of a large number of biological processes [387–389]. GAGs are water retaining polysaccharides, thus modulating the hydration and the water homeostasis in tissues [390].

GAGs can be obtained from animal sources (e.g., chondroitin sulfate is extracted from shark cartilage), albeit in heterogeneous mixtures that hampered quality control and reproducibility [391]. The heterogeneous nature of these samples is particularly problematic because the spatial orientation of the sulfate groups (sulfation code) [392–393] and the length of the polysaccharide chain affects the structural and biological features of GAGs. This became obvious during the “heparin crisis” in 2008, when batches of heparin, contaminated with oversulfated chondroitin sulfate, entered the marketplace causing the life of hundreds of people [4]. This episode underscored the urgency to develop reliable methods to access well-defined GAGs, with full control over substitution pattern and chain length. Well-defined GAGs are also valuable standards to uncover the mechanism of action of heparin-like drugs (e.g*.*, fondaparinux) [394–395].

#### Heparin and heparan sulfate

Among GAGs, heparin (H), heparan sulfate (HS), and heparin-like structures have been studied extensively due to their biological relevance [389]. HS binds to over hundred proteins depending on sulfation pattern and chain length. Generally, a minimal length (in most cases an octasaccharide) is required to trigger specific biological events, including interaction with cytokines and chemokines growth factors [396].

**Figure 17:**
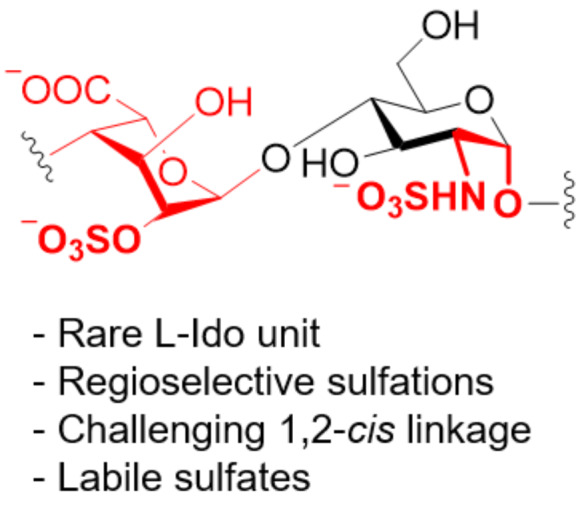
Synthetic challenges associated to the H/HS synthesis.

Due to their structural complexity, the synthesis of H/HS poses multiple challenges (Figure 17) [397]. The formation of the 1,2-*cis* linkage in the GlcN-GlcA repeating units cannot be directed with neighboring group participation. A plethora of PGs has been used to ensure or enhance α-stereoselectivity, through remote participation and/or electronic effects. In addition, orthogonal PGs should also be strategically placed to allow for the subsequent regioselective sulfation. This requirement becomes particularly challenging considering the multiple steps needed to generate the rare ʟ-idose unit. Another bottleneck arises from the lability of the sulfate groups in both acidic and basic conditions, limiting PG manipulations or functional group transformations. Thus, the removal of the PG must be carefully planned to avoid small pH fluctuations. Not last, purification and detection of highly sulfated structures is troublesome [398–399].

The significant efforts of the last 20 years resulted in synthetic methods to access various BBs for the assembly of heparin and heparan oligosaccharides. Considerable success has been obtained in making the core dimers IdoA-GlcN that can be further assembled to give longer oligomers [400–404]. Still, most methods suffered from lengthy and laborious processes (18–29 steps) and low yields that precluded scalability (Figure 16B).

**Scheme 20:**
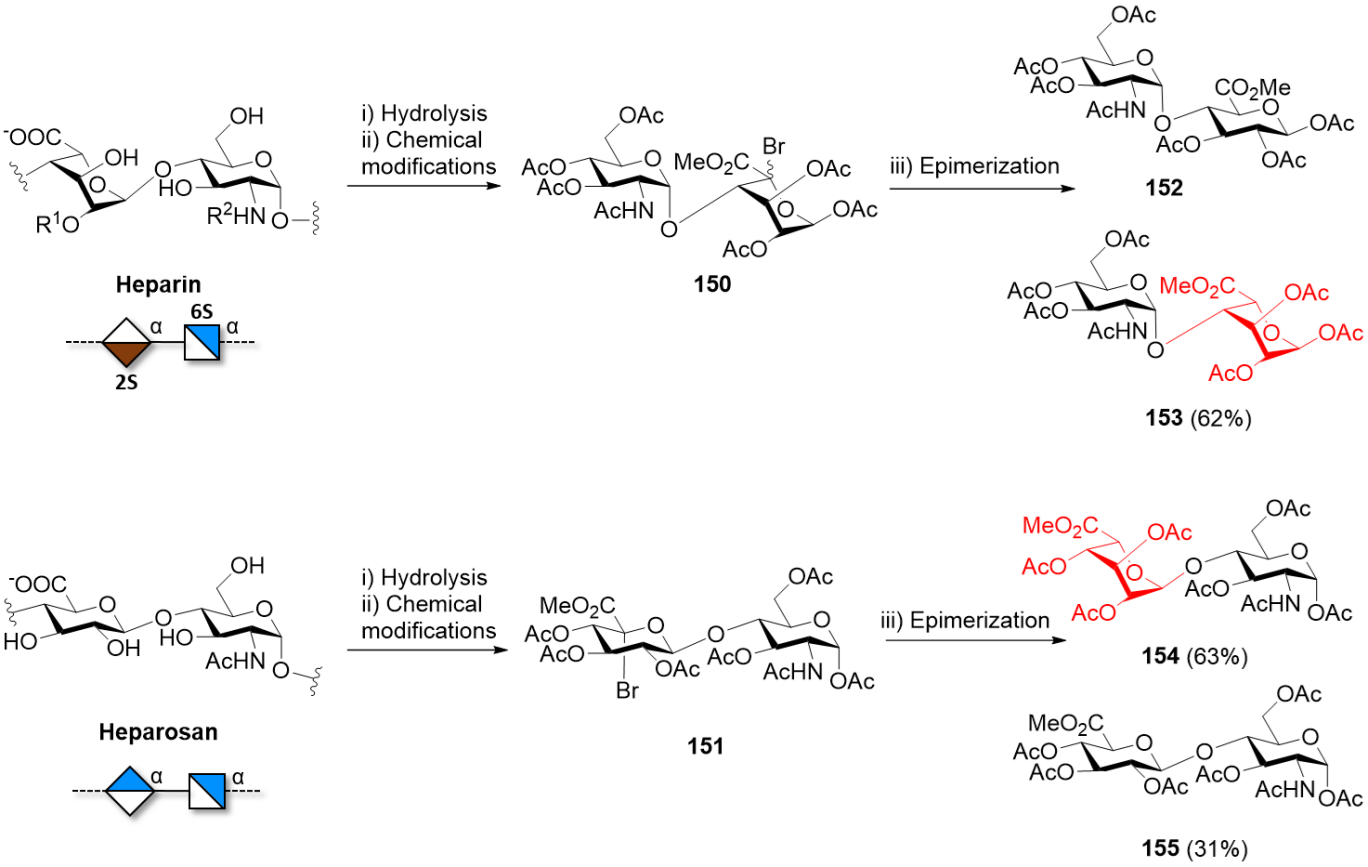
Degradation of natural heparin and heparosan generated valuable disaccharides **150** and **151** that can be further converted into four disaccharide BBs **152**–**155** to assemble well-defined structures.

Recently, a clever approach suggested the controlled hydrolysis of commercially available heparin and heparosan polymers to overcome the challenges associated with the chemical synthesis of the disaccharide cores (Scheme 20) [405]. NMR spectroscopy revealed that, after acid hydrolysis, both polymers produced one type of dimer as the major product (e.g., A-B or B-A). Epimerization strategies (i.e., GlcA→IdoA) granted access to the other two dimers present in HS. The introduction of orthogonal PGs and selective sulfation, provided a set of disaccharides in high yields (9% overall) to be used in the synthesis of well-defined heparin-like structures. To date, this scalable and efficient approach was applied to the synthesis of tetramers.

**Scheme 21:**
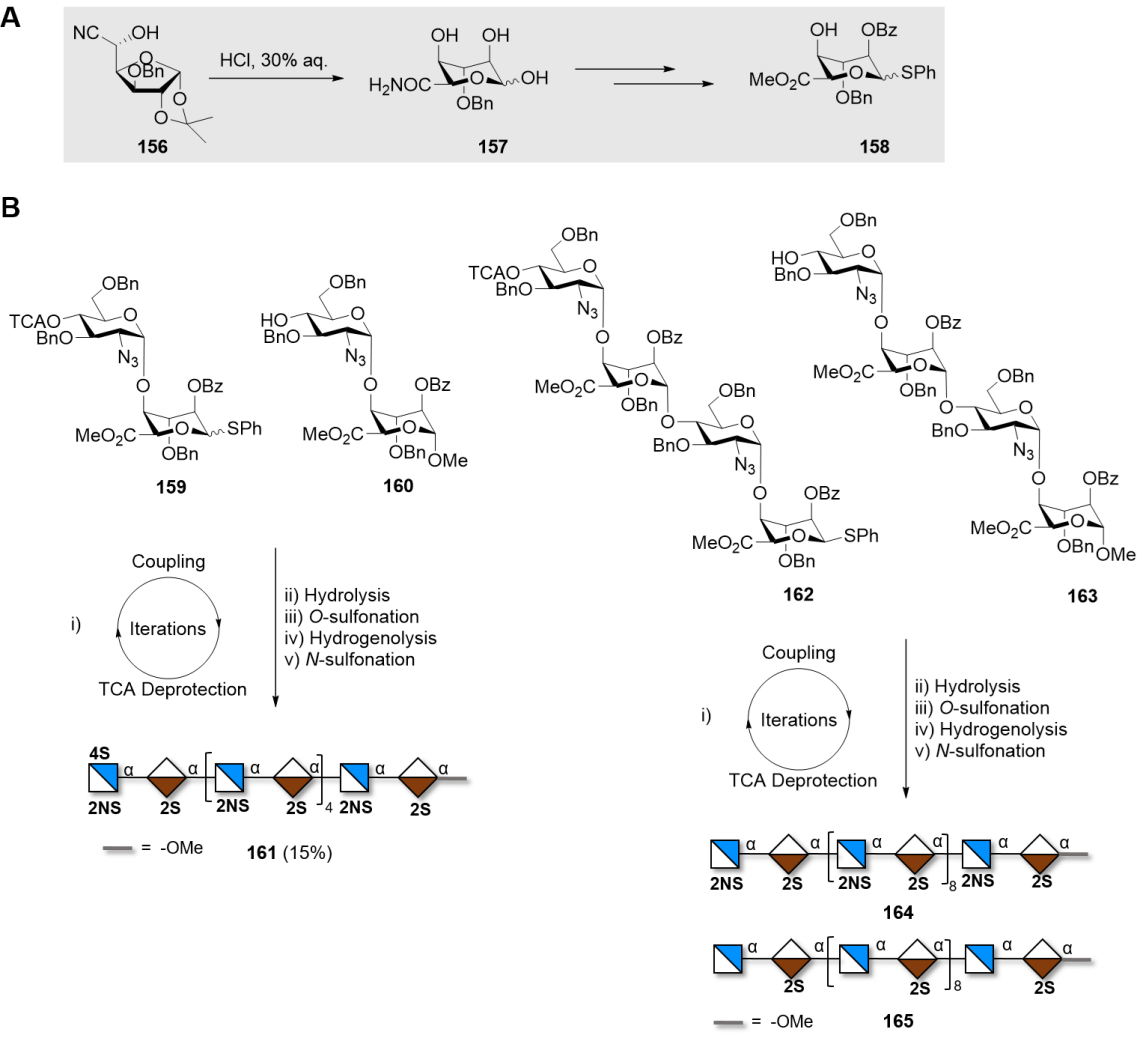
A) The one-step conversion of cyanohydrin **156** to ʟ-iduronamide **157** represent the key step for the synthesis of BB **158.** B) Synthesis of long heparin-like structures using iterative coupling strategies.

Impressive results in terms of scalability were achieved starting from the diastereomerically pure cyanohydrin **156** that was quantitatively converted to ʟ-iduronamide **157** [406]. Upon suitable PG manipulations, compound **157** was transformed into acceptor **158** that enabled the formation of the challenging α(1–4) glycosidic linkage with the 2-azidoglucoside donor (Scheme 21). The synthesis of a 12mer was achieved using iterative couplings of compound **159**. Cleavage of the ester PGs, *O*-sulfonation, azide reduction and debenzylation, followed by final *N*-sulfonation permitted the first gram-scale synthesis of a heparin-related 12mer **161** [407].

A similar iterative strategy, using tetrasaccharide BB **162** [408], led to the successful synthesis of a collection of protected HS analogues, from 16mer to 40mer [409]. The use of tetrasaccharide **162** permitted to reduce the number of purification steps after each iterative coupling and improve the overall yields. From this collection, the 20mer was further transformed into two homogeneous HS-like oligosaccharides **164** and **165** with different sulfation patterns (Scheme 21B).

**Scheme 22:**
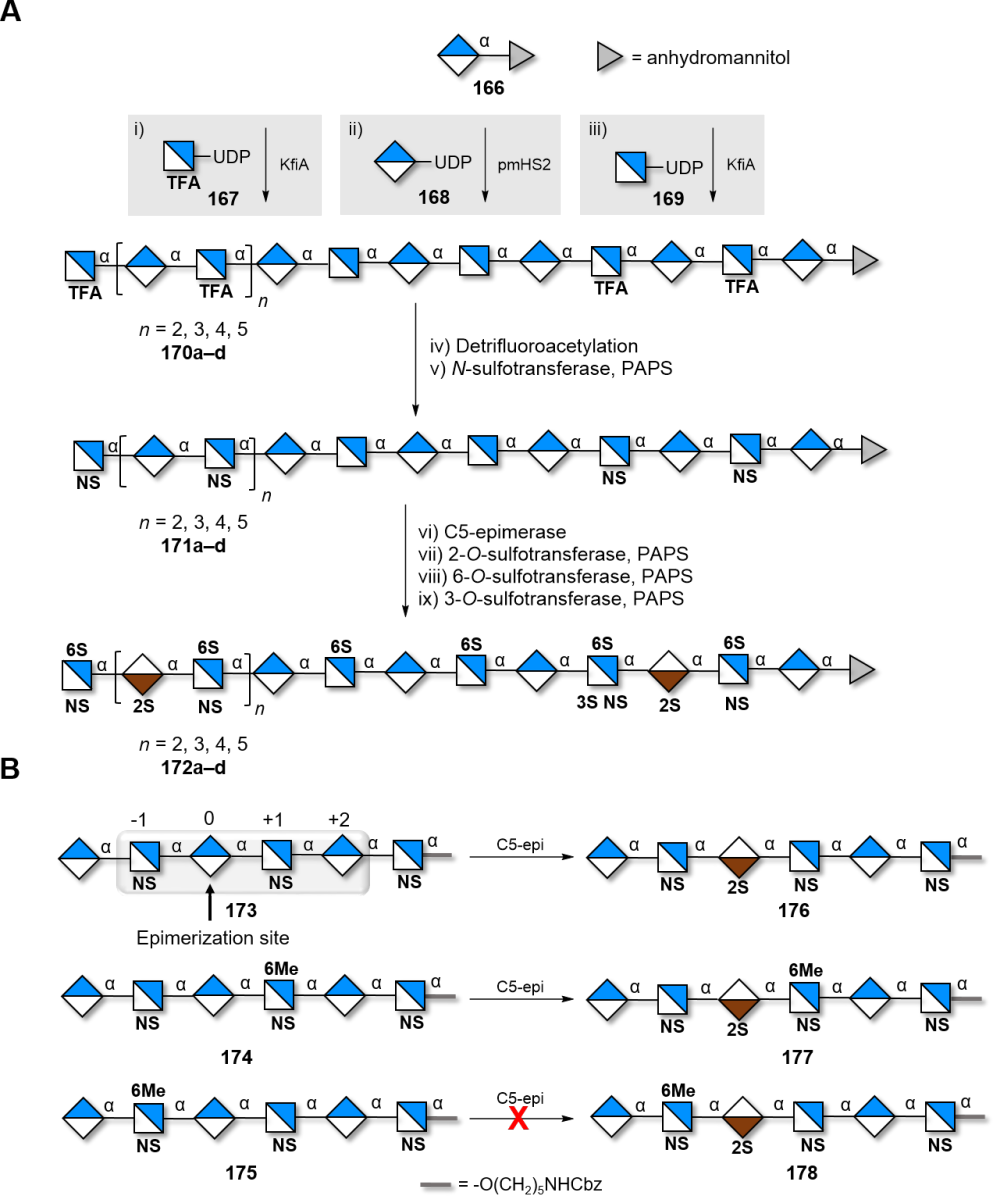
A) Chemoenzymatic synthesis of heparin structures, using different types of UDP activated natural and unnatural monosaccharides. The steps follow the in vivo biosynthesis of such compounds. B) Selective epimerization of hexasaccharides **173**, **174**, and **175** revealed how single-site methylation can affect the function of C5-epi.

Despite these impressive results, chemical methods based on repetitive coupling steps, deprotections, and purifications are extremely labor demanding. Enzymatic synthesis offers an alternative approach to shorten these lengthy procedures. Nevertheless, a purely enzymatic approach has been proven inadequate for the synthesis of long GAGs, because it generates ill-defined mixtures of oligomers with different lengths [410]. A chemoenzymatic approach could be the ideal solution to prepare well-defined heparin structures [399]. Key to a successful chemoenzymatic route is to design a sequence that resembles the in vivo biosynthesis. Bacterial glycosyltransferases (GTases) KfiA and PmHS2 are commonly used for the elongation of a starting disaccharide using UDP-GlcNAc **169** and UDP-GlcA **168** activated sugar donors [411]. *N*- and *O*-sulfotransferases (STases) catalyze the regioselective insertion of the sulfate moieties using 3’-phosphoadenosine 5’-phosphosulfates (PAPS). To facilitate the detection and the purification steps, the acceptor could be covalently attached to a UV detectable chromophore, such as *para*-nitrophenol [412] (*p*-NP), or a fluorous tag [413]. Chemical modifications, tolerated by the enzyme, were used to overcome particular bottlenecks of the purely enzymatic approach. For example, while the natural substrate for KfiA (UDP-GlcNAc **168**) proved very stable to *N*-deacetylation, the unnatural donor [414–415] UDP-GlcNTFA **167** could be efficiently *N*-deacylated under mild basic conditions, permitting subsequent regioselective *N*-sulfation. Variation on the chemical backbone were catalyzed by C5-epimerase (C5-epi), able to convert GlcA into IdoA and vice versa [416]. Recent studies revealed that C5-epi recognizes specific binding domains, promoting reversible or irreversible epimerization [417]. Following these paths, low-molecular-weight heparins (LMWH) were produced in a gram scale. Longer structures (up to 21mer, **172a–d**) were also accessible (Scheme 22A) [418]. Still, the preparation of structures containing a large number of IdoA2S-GlcNS6S remains problematic. A random epimerization may happen in the presence of C5-epi, leading to ill-defined mixtures of GlcA and IdoA in the final product. A pioneering strategy demonstrated that epimerization could be successfully controlled with the selective introduction of a 6-*O*-methyl ether on a GlcN residue (Scheme 22B) [419]. Three model hexasaccharides **173**, **174**, and **175** demonstrated how single site substitutions can be key to direct enzymatic modifications and generate knowledge on enzyme specificity.

#### Others GAGs

Chondroitin sulfate (CS) is comprised of β(1–4)-linked *N*-acetyl-ᴅ-galactosamine (ᴅ-GalNAc) and β(1–3)-linked ᴅ-glucuronic acid (ᴅ-GlcA) repeating units (Figure 16A). Dermatan sulfate, also named chondroitin sulfate B, is an analogue of CS, having IdoA instead of the GlcA repeating unit. In analogy to heparin and HS, the sulfation pattern dictates the interaction with other biomolecules resulting in important biological functions such as cell proliferation, tissue morphogenesis, and wound repair [420].

**Scheme 23:**
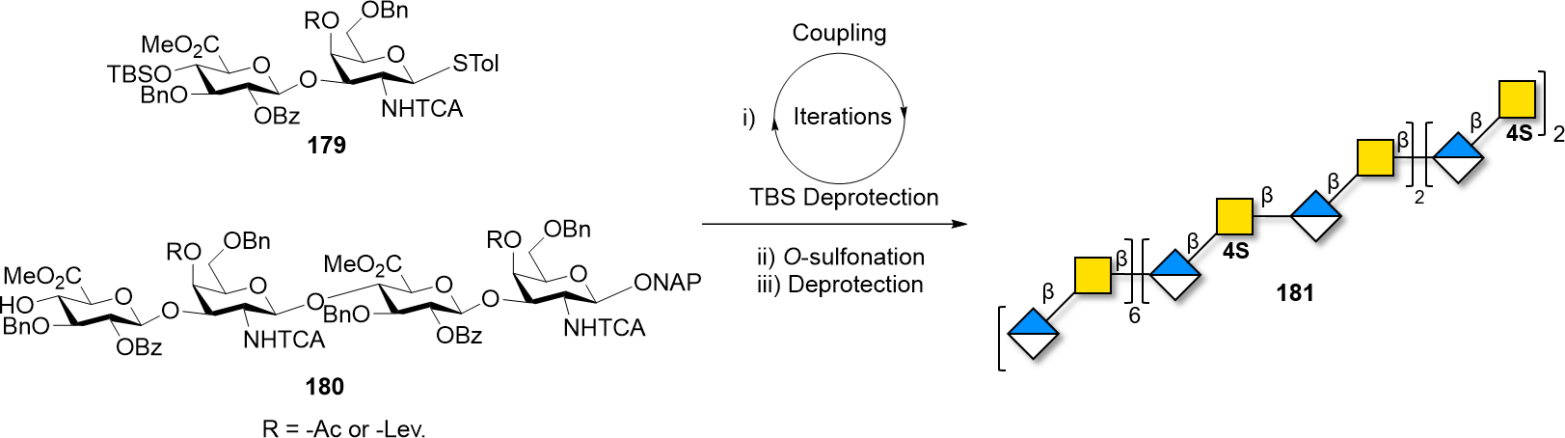
Synthesis of the longest synthetic CS chain **181** (24mer) using donor **179** and acceptor **180** in an iterative approach.

To date, the longest CS chain chemically synthesized was a 24mer [421]. To ensure stereocontrol, the nitrogen on the GalN unit was equipped with a participating PG, which can be afterwards easily removed to release the free amine. The screening of different disaccharide donors revealed that the di-4,6-*O*-acetylated GalNTFA gave the desired β-selectivity. However, the formation of a stable oxazoline side-product during the glycosylation of bigger and less reactive acceptors limited this approach. Replacement of TFA with TCA and use of Bn groups instead of the electron withdrawing esters limited the formation of the oxazoline side-product. Iterative couplings of dimer **179** allowed for the synthesis of the protected 24mer (Scheme 23). The cleavage of the Lev groups liberated the hydroxy groups that were sulfated using SO_3_·NEt_3_. Different methods were attempted for the removal of the TCA groups, which proved to be very stable. Finally, a concentrated ammonia solution permitted the cleavage of the twelve TCA moieties to give compound **181**, after hydrogenolysis.

A total of 15 CS oligosaccharides with different sulfation patterns, ranging from trisaccharides to nonasaccharides, were enzymatically synthesized to investigate their biological function [422]. The glycosyl transferase KfoC [423], extracted from the *E. coli* K4 strain, permitted to transfer both UDP-GalNAc and UDP-GlcA. The backbone could be prepared in a gram scale, thanks to the UDP-sugars obtained from cheap starting materials. This approach proved to be highly divergent, because KfoC tolerated the unnatural substrate GalNTFA, which could be chemically transformed to either an amine or an azide for further functionalization. A collection of well-defined oligosaccharides with natural and unnatural sulfation patterns were produced on multi-milligram scales.

Hyaluronic acid (HA) is the only non-sulfated GAG and is composed of alternating GlcA and GlcNAc residues connected through β(1–3) and β(1–4) glycosidic linkages, respectively (Figure 16A). HA is a major component of the extracellular matrix and is involved in many essential biological processes [424] such as cell adhesion, cell migration, and wound healing. Its mode of action is length-dependent; thus much effort has been put to obtain defined hyaluronans of different lengths. The low reactivity of the GlcA as glycosyl donor is the major synthetic challenge, generally overcome by the use of the more reactive Glc donor followed by a post-glycosylation oxidation to convert it to GlcA. Issues associated to GlcNAc concern the protection of the amino group: TCA, commonly used to direct the formation of the β-linkages through participation, often led to the formation of less reactive oxazolines. The use of TMSOTf suppressed the formation of this side product and allowed for the synthesis of a 10mer in moderate yields [425].

**Scheme 24:**
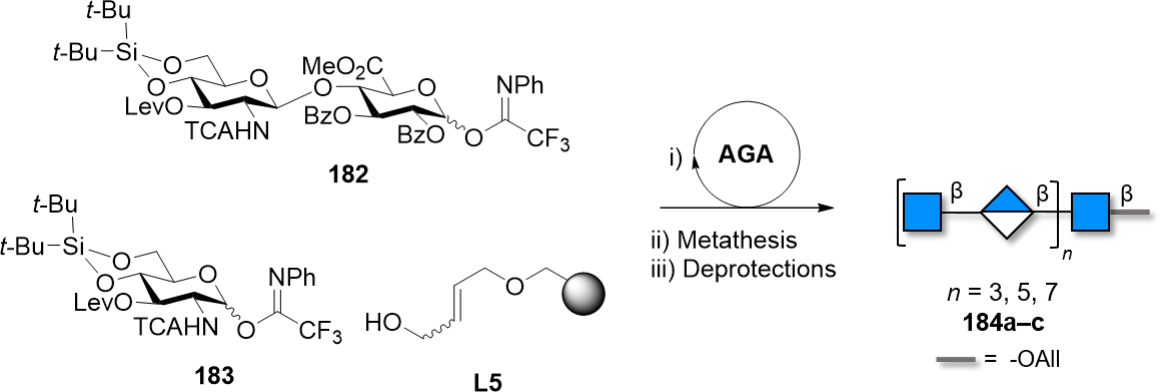
AGA of a collection of HA with different lengths. The AGA cycle includes coupling (TfOH) and Lev deprotection (hydrazine).

A collection of hyaluronans, ranging from trimers to 15mer, has been synthesized in an automated fashion on a solid-support [426]. Disaccharide **182** was prepared in a multigram scale from compound **183**. The C-4 and C-6 hydroxy groups of GlcNAc were masked as silyl ethers, providing ketals with excellent stability to acidic conditions. A first glycosylation with monomer **183** ensured high yield of the first coupling to the solid support. Iterative couplings with dimer **182** gave the protected hyaluronans with well-defined lengths that were liberated from the solid support and subjected to global deprotection to give **184a–c** (Scheme 24).

HA oligomers were prepared chemoenzymatically, using PmHAS, a unique class of bacterial enzymes that catalyze the addition of both UDP-GlcNAc and UDP-GlcA monosaccharides [427]. Short oligomers (2-4mers) were obtained after stepwise enzymatic couplings, controlling the acceptor/donor molar ratio. These oligomers served as synthons for the one-pot polymerization. HA polymers formed from short length acceptors displayed a very narrow MW distribution, as confirmed by multi-angle light scattering experiments. The same strategy was applied for the synthesis of homogeneous chondroitin polymers and other derivatives [428].

Keratan sulfate (KS) consists of a *N*-acetyllactosamine backbone (Figure 16) [429]. Different types of KS are present in the cornea, cartilage, and brain, named KS I, KS II and KS III, respectively [430]. Despite some encouraging results, the synthesis of long KS is, to date, not reported. A chemoenzymatic approach allowed for the step-by-step elongations of the backbone, but remained limited to tetrasaccharides [431]. Hexasaccharides with different sulfation pattern were obtained by AGA [432]. The low reactivity of the donor and the acceptor as the chain length increases has limited the formation of longer structures.

Overall, GAGs with well-defined composition, patterns and lengths are needed to unlock the “sulfation code” and establish structure–function correlations. Recent advances in chemical and chemoenzymatic synthesis permitted the preparation of impressive targets. Still, most procedures are lengthy and low yielding, suffering from low reactivity or poor selectivity as the polysaccharide chain grows. New automated procedures as well as highly selective enzymatic transformations should be implemented to provide valuable alternatives to naturally sourced GAGs.

## Conclusion

Exploitation of polysaccharides has always relied on mixtures of compounds extracted from natural sources. Much effort has been put to understand how sequence, length, and substitution pattern affect polysaccharides’ biological and material properties [93,221,350]. Therefore, well-defined polysaccharide samples become essential probes to establish structure–property correlations and for reproducible biological studies [93,221,350]. Polysaccharides with well-defined chemical structures can be prepared synthetically following three main approaches: i) enzymatic methods, ii) polymerization, and iii) chemical synthesis. These approaches are complementary to each other, offering advantages and limitations. Thus, in most cases, the choice of the more suitable methods depends on the desired application.

Enzymatic and chemoenzymatic methods showed enormous potential for the synthesis of some classes of polysaccharides (i.e., GAGs). The main advantages are the simple synthetic protocols (no PGs required) and the high regio- and stereoselectivity, typical of enzymes. In contrast, the high specificity of the enzymes hampers the introduction of unnatural moieties and limits the scope of these methods. To date, not all glycosidic linkages are accessible and repetitive polymers are often obtained as mixtures with a non-uniform dispersion of MW. The combination of enzymatic methods with chemical strategies (i.e., chemoenzymatic methods) offers a valuable alternative to generate precision polysaccharides [399]. Synthetically produced oligomers can be enzymatically polymerized to obtain artificial polymers with well-defined branching pattern [7]. Being able to control the polysaccharide length remains the next challenge. The inspiration could come from length-controlled biosynthetic pathways [351,354–355].

Polymerization approaches allowed growing large synthetic polysaccharides in short time and are easily scalable. However, only few types of glycosidic linkages are accessible and mixtures of different chain lengths are often generated. Recently reported methods for polymerization in confined space could improve the control over the chain length [433–435].

Chemical synthesis enabled the preparation of well-defined polysaccharides, but is labor-intensive, requiring several protected monosaccharide BBs and, to date, it suffered from poor scalability. Recently achieved total syntheses (i.e., 92mer [385] and 128mer [325]) showed that long and well-defined polysaccharides could be accessed. With automated platforms, long polysaccharides could be obtained routinely (i.e., 100mer and 151mer [278]), considerably reducing the lengthy synthetic protocols traditionally needed for solution phase methods. Still, only some classes of polysaccharides have been synthesized, often relying on the relatively easy installation of 1,2-*trans* glycosidic bonds. In contrast, the formation of multiple 1,2-*cis* glycosidic bonds remains highly challenging. New highly selective methods have been developed [167,178,285], however, only few proved to be applicable to the synthesis of long polysaccharides. Additionally, the low reactivity of particular BBs (e.g., uronic acids for the synthesis of GAGs) hampered the establishment of efficient synthetic protocols. A major limitation encountered in many reports of polysaccharide total synthesis is the decreased reactivity of large donors and acceptors. This issue is, to date, poorly predictable, due to the little knowledge on the conformation of protected glycans. Computational methods are expected to help the understanding of the reactivity for such complex molecules. While chemical ligation protocols enabled the total synthesis of proteins [436], similar methods are still lacking for polysaccharides and fragment coupling often requires broad screenings of LGs, solvents, and activation systems.

Aggregation is another major bottleneck that can drastically decrease the yield of polysaccharide synthesis and can occur either at the fully protected stage, during an intermediate stage [156–157] (i.e., semiprotected stage) or at the final deprotected stage [93,344].

Upon assembly of the protected polysaccharide target, either by polymerization or chemical synthesis, the removal of the PGs often proved to be challenging. Aggregation [93,156–157,437], degradation (both during methanolysis [93] and hydrogenolysis [294,325–326]), substituent migration [322,438] or cleavage are among the most common issues encountered. The development of better PGs that can be removed quantitatively is highly desirable. Particular effort should be put towards the development of novel N-PGs considering the many drawbacks of the currently employed ones [251,261]. Novel PGs [439] and more suitable solvent systems [440] could alleviate some of these problems.

The purification and characterization of long polysaccharides poses an additional challenge, especially for ionic polysaccharides [398,441]. As the size of synthetic polysaccharides approaches the size of macromolecules, the implementation of novel analytical and purifications techniques becomes necessary [283].

Overall, structural precision has become an important goal in polymer science as it is crucial to achieve novel properties or to fully understand complex architectures [442–443]. Automated techniques and solid-phase methods have become popular to generate polymers with full control over the sequence, length, and substitution [444–445]. These approaches, despite being time consuming and in most cases not yet scalable, represent the only amenable way to obtain structure–property correlations. Oligomers able to maintain the properties of longer polymeric structures could become a valuable target to fill the gap between small molecule and polymer science [446]. The same trend is true for polysaccharides. Well-defined polysaccharides could become essentials in biology where batch-to-batch reproducibility is imperative. Oligosaccharides with controlled lengths will be required to induce particular biological responses and automated techniques could grant access to collection of pure compounds for screening. Full control on the polysaccharide length and sequence will also be key to control the aggregation of these compounds and create nanomaterials with defined dimensions [447].
